# Whole-Genome Analysis-Based Phylogeographic Investigation of Streptococcus pneumoniae Serotype 19A Sequence Type 320 Isolates in Japan

**DOI:** 10.1128/aac.01395-21

**Published:** 2022-02-15

**Authors:** Satoshi Nakano, Takao Fujisawa, Bin Chang, Yutaka Ito, Hideki Akeda, Jiro Fujita, Yasufumi Matsumura, Masaki Yamamoto, Shigeru Suga, Kazunori Oishi, Miki Nagao, Makoto Ohnishi

**Affiliations:** a National Institute of Infectious Diseases, Tokyo, Japan; b National Hospital Organization Mie National Hospital, Tsu, Japan; c Nagoya City University, Graduate School of Medical Science, Nagoya, Japan; d Okinawa Prefectural Nanbu Medical Center & Children’s Medical Center, Okinawa, Japan; e University of the Ryukyus, Okinawa, Japan; f Kyoto University Graduate School of Medicine, Kyoto, Japan; g Toyama Institute of Health, Toyama, Japan

**Keywords:** *Streptococcus pneumoniae*, PCV, Japan, Tn*2010*, phylogeographic analysis, 19A-ST320, Taiwan19F-14, multidrug resistance, pneumococcal conjugate vaccine, whole-genome sequencing

## Abstract

After the introduction of the seven-valent pneumococcal conjugate vaccine, the global spread of multidrug-resistant serotype 19A-sequence type 320 (ST320) strains of Streptococcus pneumoniae became a public health concern. In Japan, the main genotype of serotype 19A was ST3111, and the identification rate of ST320 was low. Although the isolates were sporadically detected in both adults and children, their origin remains unknown. Thus, by combining pneumococcal isolates collected in three nationwide pneumococcal surveillance studies conducted in Japan between 2008 and 2020, we analyzed 56 serotype 19A-ST320 isolates along with 931 global isolates, using whole-genome sequencing to uncover the transmission route of the globally distributed clone in Japan. The clone was frequently detected in Okinawa Prefecture, where the United States returned to Japan in 1972. Phylogenetic analysis demonstrated that the isolates from Japan were genetically related to those from the United States; therefore, the common ancestor may have originated in the United States. In addition, Bayesian analysis suggested that the time to the most recent common ancestor of the isolates from Japan and the U.S. was approximately the 1990s to 2000, suggesting the possibility that the common ancestor could have already spread in the United States before the Taiwan 19F-14 isolate was first identified in a Taiwanese hospital in 1997. The phylogeographical analysis supported the transmission of the clone from the United States to Japan, but the analysis could be influenced by sampling bias. These results suggested the possibility that the serotype 19A-ST320 clone had already spread in the United States before being imported into Japan.

## INTRODUCTION

Streptococcus pneumoniae is one of the common pathogens in community-acquired infections and causes conditions that include pneumonia, meningitis, primary bacteremia, and otitis media. This pathogen continues to be associated with high morbidity and mortality in children; an estimated 294,000 children aged 1 to 59 months died of pneumococcal diseases globally in 2015 (1). To protect children against invasive pneumococcal disease (IPD), 7-, 10-, and 13-valent pneumococcal conjugate vaccines (PCVs) have been licensed for use in children worldwide. After the initial introduction of PCV7 in the first decade of the 2000s, an increase in serotype 19A pneumococcal infections that were not covered by the vaccine was observed in many countries (2). Serotype 19A-sequence type 320 (ST320) is one of the highly multidrug-resistant pneumococcal clones showing an increased prevalence in many counties after the introduction of PCV7, -10 and/or -13 (2, 3). This serotype 19A-ST320 clone was considered to share a ancestor with a serotype 19F-ST236 (double-locus variant [DLV] of ST320) clone that was originally recovered from a Taiwanese hospital in 1997 (4). The Taiwanese clone was named Taiwan^19F^-14 by the Pneumococcal Molecular Epidemiology Network (PMEN) (https://www.pneumogen.net/pmen/) and arose via recombination events at the capsular polysaccharide synthesis (*cps*) locus (5). Serotype 19A-ST320 isolates are generally resistant to penicillin, cefotaxime, carbapenem and macrolides, which are commonly used antibiotics in clinical settings; therefore, the spread of this clone poses a threat to global health (3, 6). While the commonly used PCV13 covers serotype 19A, understanding the dynamics of such a resistant clone is very important to promote vaccination programs in each local region because the epidemic serotypes and clones, including resistant ones, are different between countries and are changeable diachronically, which may influence the impact of pneumococcal vaccines on regional public health.

In Japan, PCV7 was licensed for use in 2010 and administered on a voluntary basis until April 2013. It was approved as a routine vaccination in April 2013 and was replaced with PCV13 in October 2013. According to several nationwide surveillance studies conducted in Japan before and after the introduction of PCVs, the increase in serotype 19A after the introduction of PCV7 was similar to that observed in other countries, and the most prevalent genotype was ST3111; ST320 was relatively rare (7–12). In brief, two nationwide surveillance studies (one for adults between 2013 and 2017 and the other for children between 2012 and 2017) demonstrated that the detection rate of 19A-ST320 among 19A isolates was nearly 0% (Fig. S1 in the supplemental material) (7, 8, 12). However, we identified several IPD cases attributable to serotype 19A-ST320 during the surveillance period. Thus, we performed whole-genome sequencing analysis to clarify the genomic characteristics and transmission route to Japan of these globally important multidrug-resistant serotype 19A-ST320 isolates that are relatively rare in Japan.

## RESULTS

### Bacterial origin and antimicrobial susceptibility.

We collected 39, 7, and 5 serotype 19A-ST320 isolates in study 1, study 2, and study 3, respectively. We analyzed five additional isolates that were sent to the National Institute of Infectious Diseases; in total, we analyzed 56 serotype 19A-ST320 isolates comprising 43 pediatric IPD, 6 pediatric non-IPD, and 7 adult IPD isolates. Thirty nine of the 49 pediatric isolates (79.6%) were from Okinawa Prefecture (Okinawa), the southernmost prefecture in Japan (Fig. S2), which consists of a number of remote islands. Similarly, six of seven adult isolates (85.7%) were from Okinawa. Thus, the distribution area of 19A-ST320 clone isolates in Japan should display an obvious pattern.

All of the 56 isolates showed intermediate resistance, resistance, or high resistance to penicillin G (PCG), cefotaxime (CTX), cefepime (CEP), meropenem (MEM), erythromycin (EM), tetracycline (TC), and trimethoprim-sulfamethoxazole (TMP-SMX) (Table 1). Similar to previous studies on serotype 19A-ST320 isolates, most of the isolates were resistant to all tested antibiotics. Notably, all tested isolates were highly resistant to PCR (MICs of >2), and 96.4% of them were highly resistant to EM (MICs of >16).

**TABLE 1:** Antimicrobial susceptibilities of serotype 19A-ST320 pneumococci in Japan

Antibiotic^*a*^	No. (%) of isolates with MIC (μg/ml) of:									
	0.5	1	2	4	8	16	32	64	128	>128
PCG	0	0	0	51 (91.1)	5 (8.9)	0	0	0	0	0
CTX	0	0	49 (87.5)	3 (5.4)	0	4 (7.1) (>8)^*b*^	NA	NA	NA	NA
CEP	0	0	52 (92.9)	0	3 (5.4)	1 (1.8) (>8)^*b*^	NA	NA	NA	NA
MEM	6 (10.7)	50 (89.3)	0	0	0	0	0	0	0	0
EM	0	0	0	2 (3.6)	0	0	0	0	0	54 (96.4)
TC	0	0	0	0	0	6 (10.7)	50 (89.3)	0	0	0
TMP-SMX	0	0	0	0	56 (100) (>4/76)^*c*^	0	0	0	0	0

a PCG, penicillin G; CTX, cefotaxime; CEP, cefepime; MEM, meropenem; EM, erythromycin; TC, tetracycline; TMP-SMX, co-trimoxazole (trimethoprim-sulfamethoxazole).

b We tested susceptibility to CTX and CEP with a MIC (μg/ml) range between 0.03 and 8.

c We tested susceptibility to TMP-SMX (1:19) with a MIC (μg/ml) range between 0.03/0.6 and 4/76.

### Whole-genome-sequencing statistics.

The average (±standard deviation) number of contigs was 139.7 (±37.1), the *N*_50_ (shortest contig length needed to cover 50% of the genome) was 69,670 (±11,397), and the mapping depth was 213.2 (±62.5) (Tables S1 and S2).

### Penicillin binding protein (PBP) type, antimicrobial resistance genes, and pilus.

Fifty-one of the 56 tested isolates carried *pbp1a*-13, *pbp2b*-11, and *pbp2x*-16, and the other five isolates carried *pbp1a*-13, *pbp2b*-11, and *pbp2x*-JP60 (*n* = 1), *pbp2x*-JP61 (*n* = 1), *pbp2x*-JP62 (*n* = 1), or *pbp2x*-JP63 (*n* = 2) (Table S1). Notably, four of the five isolates without *pbp2x*-16 (with *pbp2x*-JP60, *pbp2x*-JP62, or *pbp2x*-JP63) showed higher MICs for CTX and CEP (Tables S1 and S3), and they contained a 337SAFK motif within their *pbp2x* SXXK motifs, while all of the other 52 serotype 19A-ST320 isolates from Japan exhibited an SAMK motif within these motifs. All 56 tested isolates showed *ermB* (+), *mefE* (+), *tetM* (+), pili1 (+), pili2 (+), *folA* mutation (+), *folP* insertion (+), *ermTR* (−), and *tetO* (−).

### Recombination prediction and phylogenetic analysis.

**FIG 1:**
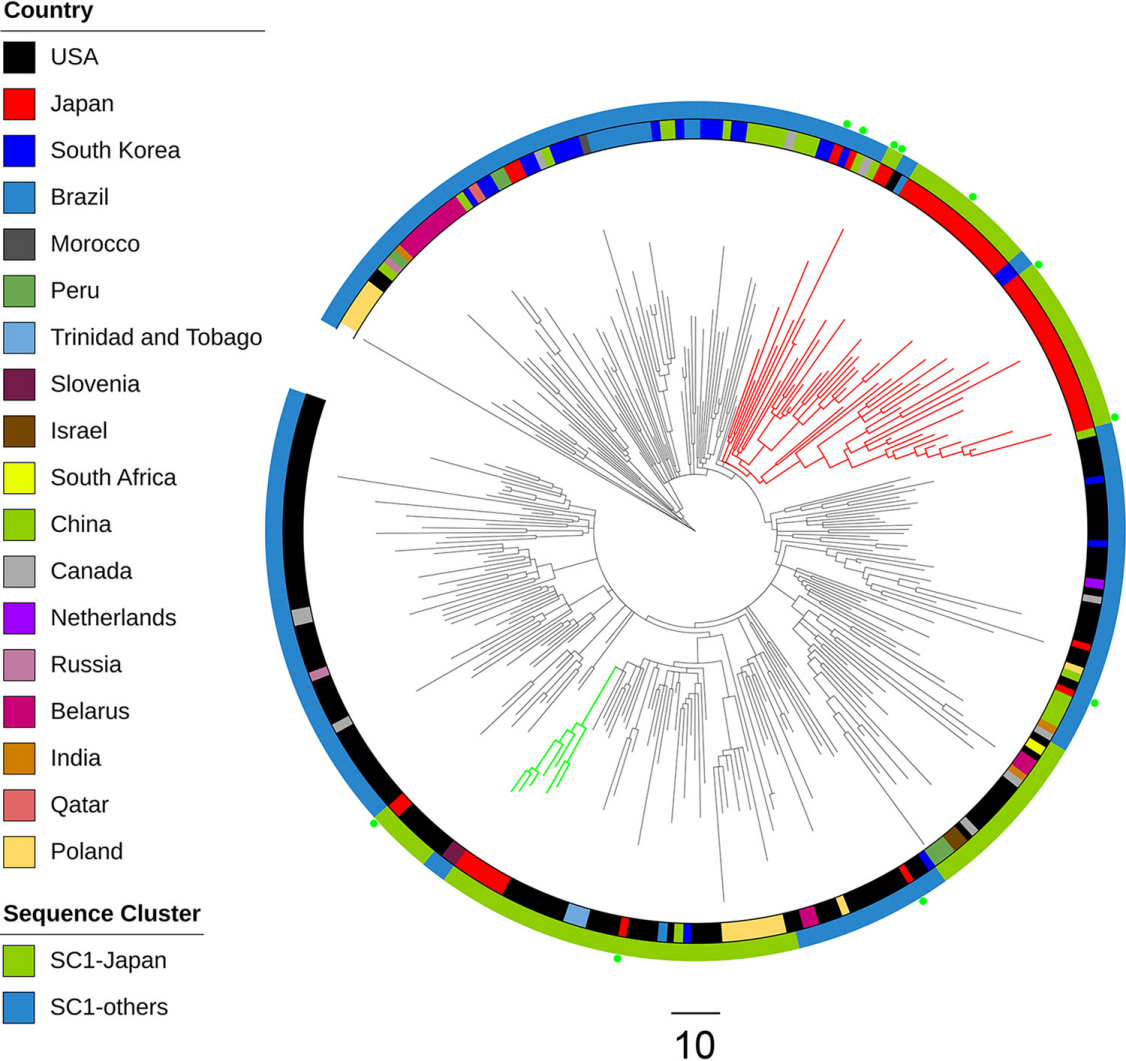
A phylogenetic tree of global serotype 19A-ST320 SC1 isolates created with Gubbins. Clusters whose branches are colored in red and light green indicate Japanese cluster 1 and Japanese cluster 2, respectively. Light green dots indicate the isolates recovered from mainland Japan. Isolates with green and blue rectangles outside the tree indicate those clustered in SC1-Japan and SC1-others, respectively, in a subsequent analysis by rhierBAPS.

We downloaded 939 GPSC1 pneumococcal genome data sets from the European Nucleotide Archive (ENA) (Supplemental Data Set S1) and generated a recombination site-censored phylogenetic tree for the isolates with the complete Streptococcus pneumoniae Taiwan19F-14 sequence as an outgroup (Fig. S3). Through rhierBAPS analysis, we obtained a total of 20 sequence clusters (SCs), and all 56 serotype 19A-ST320 isolates from Japan were clustered into SC1, which consisted of a total of 308 isolates from 18 countries. The greatest number of isolates in SC1 came from the United States (*n* = 131), followed by Japan (*n* = 56), South Korea (*n* = 25), China (*n* = 23), and Poland (*n* = 16). Isolates from other Asian countries (India [*n* = 3], Israel [*n* = 2], and Qatar [*n* = 1]) were also included in SC1. To obtain a more detailed population snapshot of SC1, we performed Gubbins analysis again for the 308 isolates. In this analysis, we obtained two subclusters that included more than five isolates from Japan, named Japanese cluster 1 and Japanese cluster 2 (Fig. 1). The larger subcluster consisted of 41 isolates from Japan, including 34 isolates from Okinawa, and 9 isolates from five other countries, and the smaller subcluster consisted of 7 isolates from Okinawa. Seven of the 11 isolates that were recovered from mainland Japan were clustered in the larger subcluster, and the other 4 isolates were clustered exclusively with each other.

We identified a total of 6,997 recombination regions in the first Gubbins analysis using all 995 global isolates. The sizes of the recombination regions ranged from 5 to 96,555 bp (median, 1,403; first quartile, 219; third quartile, 6,014), and the ratio of the number of single-nucleotide polymorphisms (SNPs) imported by recombination to those arising independently in nonrecombining regions (*r/m*) was 5.11 overall (Table S4). In each SC, *r/m* varied between 1.63 (SC9) and 9.02 (SC3).

### Tn*916*-like ICEs.

We assigned Tn*916*-like integrative conjugative element (ICE) numbers to all SC1 isolates, including all isolates from Japan. All 56 isolates from Japan harbored Tn*2010* with an *ermB* element between open reading frame 19 (ORF19) and ORF20 and a MEGA element within ORF6 (15). In the phylogenetic tree of the Tn*916*-like ICE region of the SC1 isolates, we found a total of 16 clusters that included more than one isolate with the same sequences (Fig. S4 to S6). The largest cluster consisted of a total of 191 isolates, including 37 of the 56 isolates from Japan, indicating that the Tn*916*-like ICE in SC1 was relatively well conserved; however, we found a total of 71 different types of sequences, most of which may have arisen from mutation. On the other hand, 17 branches showed obviously longer lengths than the others, indicating that recombination events are likely to occur within and/or across the Tn*916*-like ICE (i.e., Tn*2010* in this case).

### Bayesian analysis.

**FIG 2:**
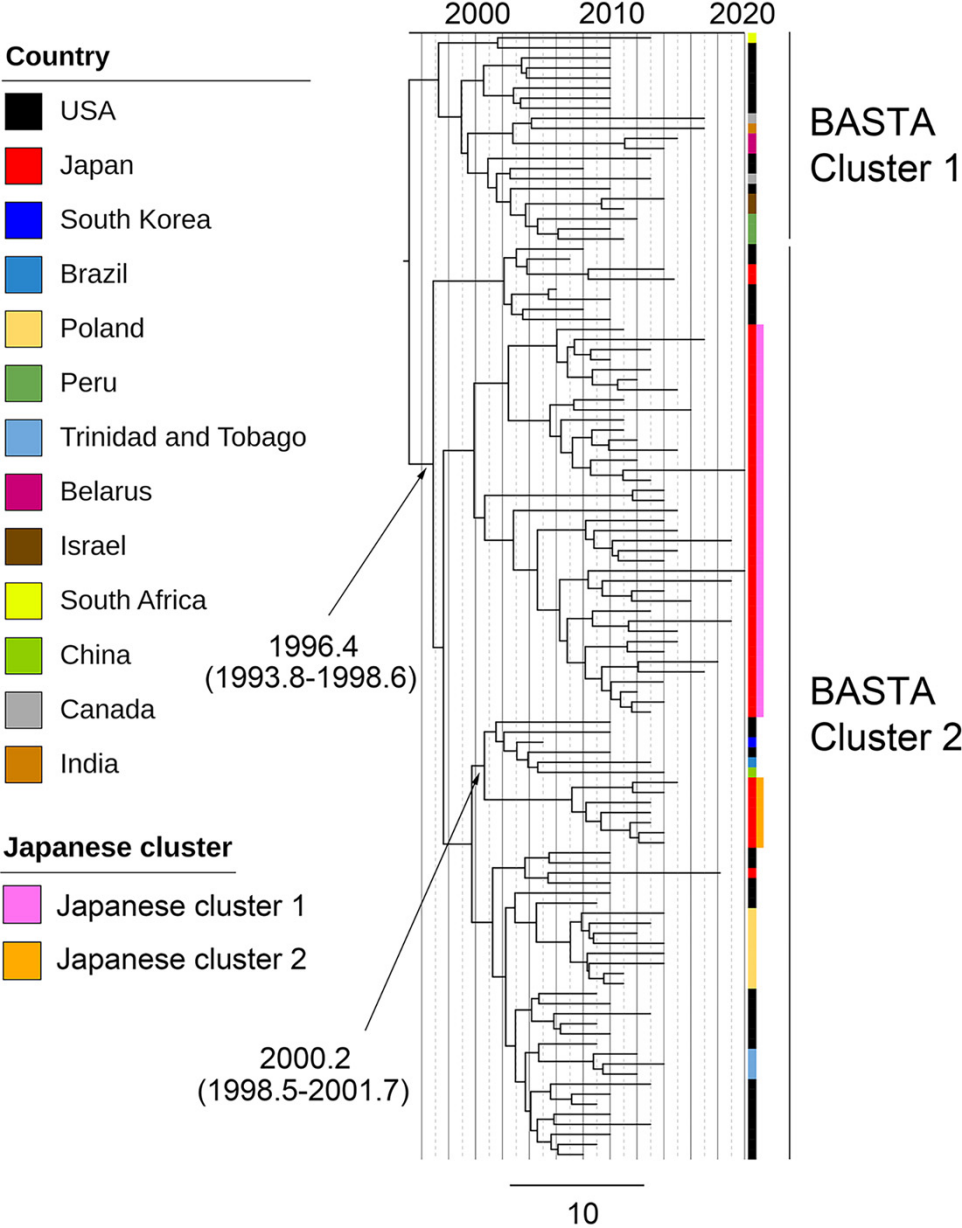
Maximum clade credibility tree of group 1 isolates in SC1 created using BEAST. The black arrow indicates the estimated date when the Japanese clades (Japanese cluster 1 and cluster 2 from Fig. 1) diverged from the most recent common ancestor (MRCA) with isolates from another country.

To reduce the computational intensity, we divided the SC1 isolates into two groups (SC1-Japan and SC1-others) using rhierBAPS, resulting in 49 of the 56 isolates from Japan being subclustered into SC1-Japan. Therefore, we estimated the time of the most recent common ancestor (tMRCA) of the serotype 19A-ST320 isolates from Japan in SC1-Japan with respect to those in the United States by using BEAST. In this analysis, we identified two clusters (equal to Japanese cluster 1 and Japanese cluster 2) that consisted of more than five isolates from Japan; the tMRCA (95% highest posterior density [HPD]) estimates for the clusters were 1996.4 (1993.8 to 1998.6) and 2000.2 (1998.5 to 2001.7), respectively (Fig. 2 and Fig. S7).

**FIG 3:**
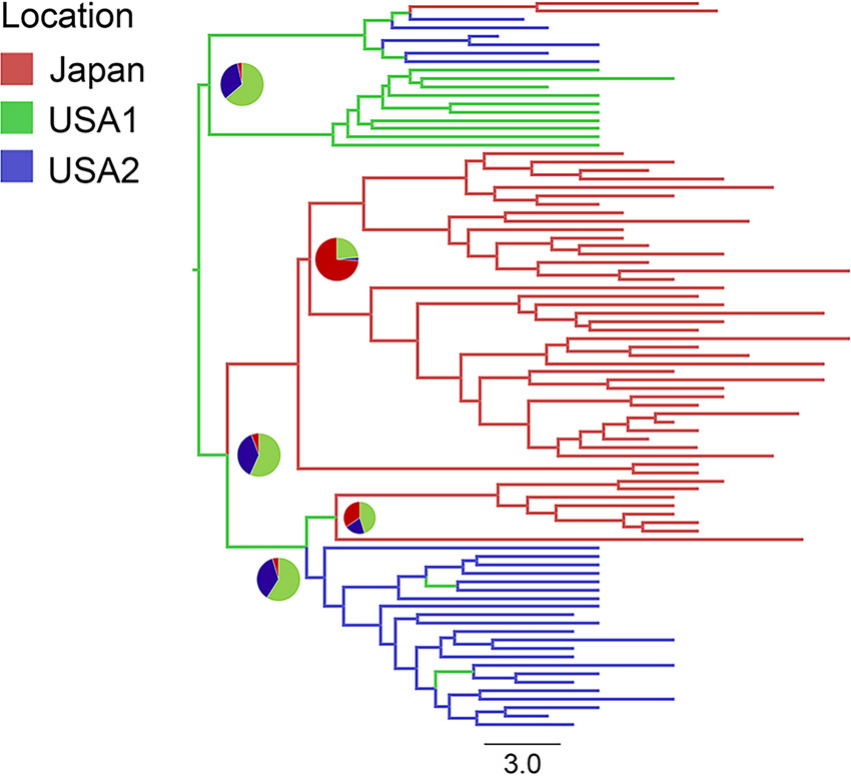
Maximum clade credibility tree with transmission history inferred from the Japanese and U.S. isolates that were clustered in SC1 group 1 using BASTA. Branch colors indicate the inferred cluster locations at the nodes. Pie charts show the posterior distribution of locations inferred at major nodes.

For the asymmetric discrete trait phylogeographic modeling analysis, we analyzed 292 isolates from 9 counties from which five or more isolates were included in SC1. We found 12 statistically supported (Bayes factor [BF] of >3) routes of transmission between nine countries (Table 2). The highest support was obtained for transmission from the United States to Canada (BF of 231,317.7), followed by transmission from the United States to Japan (BF of 65,534.8). The transmission routes to Japan that were supported by a BF of >3 were transmissions from the United States (BF of 65,534.8) and from South Korea (BF of 255.5). The transmission route from the United States to South Korea was also supported with a BF of 61.0. For the BASTA analysis, we found that the USA1 isolates (i.e., USA isolates in BASTA cluster 1 in Fig. 2) could be ancestral to the Japanese isolates in SC1-Japan (Fig. 2, Japanese cluster 1 and Japanese cluster 2 isolates; Fig. 3; Fig. S8).

**TABLE 2:** Transmission routes supported with a Bayes factor of >3 in the phylogeographic analysis

Transmission route		Bayes factor
From:	To:	
USA	Brazil	19.5
USA	South Korea	61.0
China	Canada	84.2
USA	Belarus	97.2
South Korea	Japan	255.5
South Korea	Brazil	391.2
USA	Peru	418.8
USA	China	495.3
South Korea	China	515.2
USA	Poland	9,923.3
USA	Japan	65,534.8
USA	Canada	231,317.7

## DISCUSSION

We conducted a whole-genome-sequencing analysis to reveal the origin and dynamics of the S. pneumoniae serotype 19A-ST320 clones recovered in Japan by comparing their genomic data to those of global isolates from the GPSC database and other data sets, mainly from Asian countries.

The antimicrobial susceptibility pattern of serotype 19A-ST320 isolates from Japan was similar to that of the isolates from other countries, especially in SC1 (Fig. S3); the isolates were resistant or exhibited a high level of resistance to PCG. Notably, five isolates from Japan exhibited other *pbp2x* types, *pbp2x*-JP60, *pbp2x*-JP61, *pbp2x*-JP62, or *pbp2x*-JP63, and most of them showed a high level of resistance to cephalosporins; four of the five isolates (with the exception of one isolate carrying *pbp2x*-JP61) showed higher resistance to CTX and CEP than isolates carrying *pbp2x*-16. All of the *pbp2x*-carrying isolates with higher resistance showed the substitution of 337SAMK to SAFK in the *pbp2x* 337SXXK motif, indicating that this substitution causes higher cephalosporin resistance in serotype 19A-ST320 pneumococci. This resistance mechanism causing isolates carrying *pbp1a*-13 to show an increased third-generation cephalosporin MIC, mediated by changes in the *pbp2x* SXXN motif, was also reported in a previous study for a serotype 15A-ST9084 clone carrying *pbp1a*-13 (14).

In Japan, serotype 19A-ST320 isolates were mainly detected in Okinawa. Previous studies also revealed this distribution; in study 1 (a pediatric IPD surveillance study conducted in 10 of 47 prefectures between 2008 and 2013), 38 of 56 serotype 19A isolates (67.9%) were identified as ST320 in Okinawa, while only 1 of 122 (0.8%) was identified as ST320 outside Okinawa (the main STs of serotype 19A in mainland Japan were ST3111 and ST2331). In addition, in study 2 (an adult IPD surveillance study conducted in 10 prefectures between 2013 and 2020), 6 of 19 serotype 19A isolates (31.6%) were ST320 in Okinawa, while 1 of 166 serotype 19A isolates (0.6%) was ST320 in prefectures other than Okinawa. Therefore, we postulated that the clone was imported to Okinawa from other countries and then spread to mainland Japan. To clarify the international and intranational routes of spread of the clone, we conducted a phylogenetic analysis in this study. Phylogenetic tree construction using serotype 19A-ST320 isolates in Japan and all global isolates revealed that serotype 19A-ST320 isolates from Japan were clustered into a single sequence cluster (SC1), suggesting that the isolates from Okinawa and mainland Japan were genetically related, even though they were geographically separated from each other. Subsequent analysis using only the SC1 isolates revealed that the isolates in Okinawa were separated into multiple subclusters, suggesting the clone was imported into Okinawa on several different occasions and then spread regionally. In addition, among the isolates from mainland Japan, some clustered with Okinawa isolates and some clustered separately, indicating that there were two modes of transmission to mainland Japan: transmission associated with the spread in Okinawa and transmission from Okinawa or other regions whose isolates were included in SC1 but were not associated with the spread.

We conducted two phylogeographic analyses, an asymmetric discrete trait phylogeographic modeling and a Bayesian structured coalescent approximation using BASTA, because the former method suffers from sampling bias; however, the latter method could not be used to analyze all SC1 isolates due to high computational costs. In the asymmetric discrete trait phylogeographic modeling, BFs of >100 indicate decisive support, BFs of 30 to 100 indicate very strong support, BFs of 10 to 30 indicate strong support, and BFs of 3 to 10 indicate substantial support for a model (41). Therefore, the BF value of more than 60,000 that supported the transmission route from the United States to Japan was extremely robust. In addition, we believe that the support for the transmission route from the United States to Canada based on the highest BF value (231,317.7) showed the robustness of this analysis. In the BASTA analysis, we found that U.S. isolates in SC1-Japan (Fig. 1) could be ancestral to Japanese isolates, but we could not include isolates from other countries in SC1 due to the computational costs. Considering the results of the phylogeographic analysis and the fact that transmission routes from the United States to Peru, China, and Poland were supported with high BF values (>200), the clustered isolates from the United States may have circulated around geographically distant countries that were included in SC1. Croucher et al. demonstrated similar free intercontinental transmission of PMEN1 (also known as Spain23F-1) clones between North America, Southeast Asia, and Eastern Europe in a whole-genome-analysis-based phylogenetic investigation (16). On the other hand, we have to note that discrete trait analysis, in general, suffers from various biases, such as sampling bias (17, 18). In fact, the transmission route from South Korea to Japan was also supported with a BF of 255.5. Therefore, we have to interpret the results carefully, as most of the phylogeographical analyses suggest that serotype 19A-ST320 isolates in Japan seemed to have ancestors from the United States.

In the case of the transmission of SC1 from the United States to Okinawa, we anticipated that the transmission was mediated by American people living in Okinawa. Okinawa was governed by the United States after World War II and was returned to Japan in 1972. Thereafter, many American people continued to live in Okinawa because many United States military bases remained there. In addition, the high population density in the Okinawa region may be one of the reasons contributing to the spread of the 19A-ST320 clone in Okinawa; the main island of Okinawa has the 7th highest population density among the 47 prefectures of Japan. A previous study suggested that a high population density is one of the risk factors for an increase in the antibiotic resistance rate of pneumococci (39). Thus, a high population density could facilitate the spread of the resistant clone in the region. This could be a factor contributing to the frequent isolation of the 19A-ST320 clone only from Okinawa within Japan, even though there are many American people living in areas other than Okinawa in Japan. Serotype 19A-ST3111 isolates that were the most prevalent isolates of serotype 19A in mainland Japan were not as resistant as serotype 19A-ST320 isolates found in Japan. The resistance rate to PCG of the former was approximately 50% (7), while that of the latter was 100%; thus, the resistance rate was not likely to be associated with the prevalence of the two clones. Therefore, the factors that prevented the spread of the more resistant clone in mainland Japan remain unclear; further studies are needed to uncover factors like population dynamics that could change the prevalence of pneumococcal clones and the genetic features that influence the tendency of clonal spread.

The divergence time estimation demonstrated that the serotype 19A-ST320 clone from Japan diverged in the 1990s before the introduction of PCV7. This result was consistent with the results from other epidemiological studies published previously (20, 21), in which 19A-ST320 pneumococci were isolated in Southeast Asia in the late 1990s. In addition, a previous study suggested on the basis of Bayesian coalescent analysis that the Taiwan19F-14 clone arose in approximately 1987 (5); therefore, this clone was estimated to have been imported into Okinawa relatively soon after its emergence, implying that the global spread of this clone was rapid, particularly in the United States, after its emergence in approximately the 1980s. In addition, the result that the Japanese cluster 2 isolates in the maximum clade credibility tree in Fig. 2 shared a most recent common ancestor not only with the United States isolates but also with Chinese, Brazilian, and South Korean isolates may support this estimation.

We have to note one limitation of this study. While we suggested the possibility of American people in Okinawa transporting the resistant clone from the United States to Okinawa, there is no direct evidence of the transmission mode. Our surveillance study did not include patient nationality data; therefore, we could not prove the genetic association of the isolates with patient nationalities. To obtain a higher-resolution snapshot of the global dynamics of pneumococci, including resistant clones, further studies are needed. In addition, as we mentioned above, we have to note the accuracy of the transmission route prediction based on Bayesian analysis: unsampled and undersampled locations could influence the results. In general, the locations from which isolates were oversampled tend to be identified as an origin of the clone, and those from which isolates were not sampled are not able to be identified as the origin in discrete trait analysis. Therefore, the supported transmission route of 19A-ST320 isolates from the United States to Japan was identified based solely on the locations sampled in this study.

In conclusion, we analyzed 19A-ST320 isolates that were recovered mainly in Okinawa, a remote island region in Japan, between 2008 and 2020. The isolates showed an antibiotic susceptibility pattern similar to that of isolates from other countries, and a change in the *pbp2x* 337SXXK motif from SAMK to SAFK caused higher resistance to CTX and CEP. Phylogenetic analysis suggested the existence of several subclusters of serotype 19A-ST320 isolates that appeared to have been imported at different times. Through Bayesian analysis, we identified a strongly supported transmission route of the clone in Okinawa from the United States, and the isolates in Okinawa appeared to diverge in the 1990s. These results suggested that the emergence of the globally spreading multidrug-resistant serotype 19A-ST320 clone on remote islands of Japan might be caused by transmission not from geographically related Southeast Asian regions but from the American people who were visiting the region and living there.

## MATERIALS AND METHODS

### Isolates.

We analyzed serotype 19A-ST320 isolates that were collected in three nationwide pneumococcal surveillance studies in Japan. The first was a pediatric IPD surveillance study conducted in 10 of 47 prefectures between 2008 and 2013 (study 1) (10). The second was an adult (older than 15 years of age) IPD surveillance study conducted in 10 prefectures between 2013 and 2020 (study 2) (11, 12). The third was a pediatrics IPD and non-IPD surveillance study conducted in 43 prefectures between 2012 and 2017 (study 3) (7, 8). In addition, we analyzed five serotype 19A-ST320 isolates that were sent to the National Institute of Infectious Diseases, Japan, from regional institutes in Japan between 2009 and 2013.

These studies were reviewed and approved by the Ethics Committee of Mie Hospital and the National Institute of Infectious Diseases (NIID).

In each study, serotyping was performed using pneumococcal typing antisera (Statens Serum Institut, Copenhagen, Denmark), and STs were determined following standard methodology described previously (22). We performed susceptibility testing for penicillin G (PCG), cefotaxime (CTX), cefepime (CEP), meropenem (MEM), erythromycin (EM), tetracycline (TC), and co-trimoxazole (trimethoprim-sulfamethoxazole [TMP-SMX]) by using the broth microdilution method following the 2015 Clinical and Laboratory Standards Institute (CLSI) guidelines (23). For PCG, susceptibility, intermediate resistance, resistance, and high resistance were defined (in μg/ml) as ≤0.06, 0.12 to 1.0, 2.0, and >2, respectively. For CTX, susceptibility, intermediate resistance, resistance, and high resistance were defined as ≤0.5, 1.0, 2.0, and >2, respectively. For MEM, susceptibility, intermediate resistance, resistance, and high resistance were defined as ≤0.25, 0.5, 1.0, and >1, respectively. For EM, susceptibility, resistance, and high resistance were defined as ≤0.25, 0.5 to 16, and >16, respectively. For the other tested antibiotics (CEP, TC and ST), we used the 2015 CLSI criteria for susceptibility, intermediate resistance, and resistance.

### Whole-genome sequencing.

We extracted total genomic DNA from the tested isolates using a QIAamp DNA minikit (Qiagen, Hilden, Germany). We employed the Nextera XT DNA library preparation kit (Illumina, San Diego, CA, USA) to prepare libraries for sequencing. We multiplexed and sequenced the samples with the Illumina NextSeq 500 system via 300 cycles (2 × 150-bp paired-end reads). We obtained draft genome data for four isolates using the MiSeq system with 600 cycles (2 × 300-bp paired-end reads) in a previous study (24).

### PBP typing, identification of resistance genes, and pilus detection.

We performed PBP typing (3, 25, 26), resistance gene identification, and pilus detection as described previously (14, 24, 27). In brief, after read trimming, we assembled the obtained sequence reads and extracted the *pbp1a*, *pbp2b*, and *pbp2x* regions from the contigs using BLAST+ version 2.6.0 (28). The type numbers originated from previously published Centers for Disease Control and Prevention PBP types (26). We detected the *ermB*, *ermTR*, *mefA*, *mefE*, *tetM*, *tetO*, *rrgA-1* (pili1), and *pitB-1* (pili2) genes and searched for mutations and insertions/deletions within the *folA* and *folP* genes in the assembled contigs using BLAST+. In addition, we assigned Global Pneumococcal Sequence Cluster (GPSC) numbers (29) using Pathogenwatch (https://pathogen.watch/).

### Phylogenetic tree construction and phylogeographic analysis.

To clarify the genetic relationships between global serotype 19A-ST320 isolates and our samples, we downloaded the draft genome data of 885 GPSC1 isolates published by Gladstone et al. (29) because all 19A-ST320 isolates collected in Japan belonged to GPSC1. Additionally, we downloaded data from public databases for 7 isolates from China (30, 31), 25 isolates from South Korea (32), 11 isolates from Myanmar (33) and 10 isolates from Canada. When only assembled contigs were available, we generated 2 × 150-bp paired-end reads using BBmap (https://sourceforge.net/projects/bbmap/). We mapped the sequence reads to the complete Streptococcus pneumoniae Taiwan19F-14 sequence (GenBank accession no. NC_012469.1) using Burrows-Wheeler Aligner version 0.7.17 (34).

First, we created a recombination site-censored maximum-likelihood tree of all global isolates and our isolates from Japan using Gubbins version 2.4.1 (35). Second, we determined the number of genetically distinct clusters among the filtered SNP sequences with Gubbins using rhierBAPS version 1.1.3 (36). Third, we reconstructed a tree and obtained the dates of the ancestors or nodes of clades that included serotype 19A-ST320 isolates from Japan using BEAST version 1.10.4 (37). Finally, we performed two phylogeographic analyses: one was an asymmetric discrete trait phylogeographic modeling for SC1 isolates using a Bayesian stochastic search variable selection framework (38), and the other was a Bayesian structured coalescent approximation for SC1-Japan isolates using BASTA (17). For the asymmetric discrete trait phylogeographic modeling, we used all isolates in SC1 if five or more isolates were sampled from each country. In the BASTA analysis, we analyzed all SC1-Japan isolates from Japan (*n* = 49) and the United States (*n* = 38). In this analysis, we divided the United States isolates into two groups following an arbitrary branch length cutoff, which generated two BASTA clusters (i.e., BASTA cluster 1 and BASTA cluster 2) in the tree in Fig. 2. Isolates from the United States that clustered into BASTA cluster 1 were named USA1, and those that clustered into BASTA cluster 2 were named USA2. Details of the Bayesian analysis are shown in the supplemental material.

### Tn*916*-like ICEs.

To determine the structure of Tn*916*-like integrative conjugative elements (ICEs) in serotype 19A-ST320 pneumococci in Japan, we extracted the corresponding sequence regions from the assembled contigs using BLAST+ and SAMtools version 1.9 (19). We manually assigned transposon numbers to the tested isolates using ACT version 18.1.0 (40). Next, we generated a maximum-likelihood tree of the Tn*916*-like ICE regions using RAxML-NG version 0.9.0 (13) to assess the dynamics and clonality of the jumping elements.

### Data availability.

Sequence data have been deposited in the DNA Data Bank of Japan (DDBJ) under accession numbers DRR277768 to DRR277819.
