# Supplementary material for: Whole-Genome Analysis-Based Phylogeographic Investigation of Streptococcus pneumoniae Serotype 19A Sequence Type 320 Isolates in Japan
Source: Antimicrob Agents Chemother. 2022 Feb 15;66(2):e01395-21. doi: 10.1128/aac.01395-21 (PMC8846463; doi:10.1128/aac.01395-21)
Supplement: Supplemental file 1 — Supplemental material. Download aac.01395-21-s0002.pdf, PDF file, 1.1 MB [file aac.01395-21-s0002.pdf]

Whole-genome analysis-based phylogeographic investigation of *Streptococcus pneumoniae* serotype 19A sequence type 320 isolates in Japan.

Supplementary materials

Supplementary Methods and Results

Isolates and sequencing

We obtained draft genome data of serotype 19A-ST320 isolates recovered in Japan between 2008 and 2020 that were collected through three nationwide pneumococcal disease surveillance studies (1-4), one in adults and the other two in pediatric patients. We analyzed a total of 56 isolates from Japan, including 45 isolates from Okinawa Prefecture. Among the 45 isolates, 39 were recovered from pediatric patients. The other 11 isolates were collected from nine of the 46 prefectures in mainland Japan.

We extracted total genomic DNA from the tested isolates using a QIAamp DNA Mini Kit (QIAGEN, Hilden, Germany). We used the Nextera XT DNA Library Preparation Kit (Illumina, San Diego, CA, USA) for library preparation. We multiplexed and sequenced the samples of 52 isolates on an Illumina NextSeq 500 system over 300 cycles (2 × 150-bp paired-end). For the other 4 isolates, we obtained sequences using the MiSeq platform via 600 cycles (2 × 300-bp paired-end).

Trimming and assembly

After obtaining read data, we trimmed adapter and index sequences using fastp version 0.20.1 (5) with the following options: -e 25 -n 5 -t 1 -T 1 -l 25 -g -z 4. We used SPAdes version 3.13.1 (6) for assembly in careful mode with k-mers of 21, 33, 45, 57, 69, 81, 93, 105, 117 and 127. The quality of the assembly was evaluated with QUAST version 5.0.2 (7).

PBP typing, resistance gene detection and pilus typing

For PBP typing, we extracted the *pbp1a*, *2b* and *2x* regions from the *S. pneumoniae* G54 reference sequence (GenBank accession No. CP001015.1). We constructed a BLAST database for the three gene regions and extracted corresponding sequences from samples using BLAST+ version 2.6.0 (8). We assigned PBP type numbers to the extracted sequences; the type numbers originated from the Centers for Disease Control and Prevention PBP gene type database (https://www.cdc.gov/streplab/pneumococcus/mic.html, last updated June 2016). We detected the *ermB*, *ermTR*, *mefA*, *mefE*, *tetM*, *tetO*, *rrgA-1* (pili1), and *pitB-1* (pili2) genes and searched for mutations and insertions/deletions within the *folA* and *folP* genes in the assembled contigs using BLAST+ version 2.6.0. We followed the detection criteria for the genes published previously (9).

Phylogenetic analysis using Gubbins

We generated recombination site-censored phylogenetic trees using Gubbins version 2.4.1 (10). To create input files for Gubbins, we mapped trimmed reads to the *Streptococcus pneumoniae* Taiwan19F-14 reference sequence (GenBank accession no. NC_012469.1) using Burrows-Wheeler Aligner version 0.7.17 (11) with the -aM option.

SNP calling and the removal of duplicate reads and indels were conducted using GATK version 4.1.9 (12) following GATK Best Practices Workflows (https://gatk.broadinstitute.org/hc/en-us/sections/360007226651-Best-Practices-Workflows). In addition, we removed large indels that were detected using Pilon version 1.23 (13) with default options. Variant filtration was performed using GATK, and consensus sequence FASTA files were created using VCFtools version 0.1.16 (14). Gubbins analysis was performed with the standard parameters. We obtained sequence clusters (SCs) using rhierBAPS (15) with max.depth=2. With the n.pop parameter set at 20 or 30, we obtained 20 SCs in each case at clustering level 2; therefore, we set the parameter at 30 and proceeded with the analysis. For each SC that consisted of five or more than 10 isolates, we performed Gubbins analysis using only the isolates in each SC to obtain more accurate recombination sites within the cluster.
The average recombination rate (*r/m*) was calculated from the data obtained with Gubbins. Ancestral recombination events that occurred once and spread in the cluster via clonal descent were counted once.

Tn*916* analysis
We extracted the Tn*916*-like ICE regions of each isolate with BLAST+ version 2.6.0 using the *E. faecalis* DS16 transposon Tn*916* sequence (GenBank accession No. U09422.1) as a reference sequence. We visually compared the sequences to those in a previous publication (16) using ACT version 18.1.0 (17). To generate a phylogenetic tree, we mapped the trimmed reads to a reference sequence of Tn*2010* (GenBank accession No. AB426620.1). The mapping process was the same as that employed for Gubbins analysis. Using the ML model suggested by ModelTest-NG version 0.1.7 (18), we created an ML tree using RAxML-NG version 0.9.0 (19) with 100 bootstrap replicates.

Bayesian analysis
We estimated the time of the most common ancestor (TMRCA) of the clone that included 19A-ST320 isolates from Japan using BEAST version 1.10.4 (20). To reduce the computational intensity, we performed clustering with rhierBAPS at level 1, resulting in the division of SC1 isolates into two groups (SC1-Japan and SC1-others in Figure 2). We subsequently analyzed SC1-Japan isolates because 50 of the 56 isolates from Japan clustered into group-1.
We reran the Gubbins analysis for the SC1-Japan isolates and removed recombination sites to create an input file for BEAST analysis. SNP site alignment files were created using snp-sites version 2.5.1 (21). To identify sequences whose genetic divergence and sampling date were incongruent, we used TempEst version 1.5.3 (22). The analysis of the best-fitting root mean square of the heuristic residual after removing isolates that appeared to be problematic sequences showed that the correlation coefficient (R^2) of SC1-Japan was 0.3377. The model was selected through comparisons of the marginal likelihood using path sampling and stepping stone-based marginal likelihood estimation for a strict clock and an uncorrelated relaxed clock in a molecular clock model or a constant size and Bayesian Skygrid in a tree prior model. For each setting, we used default priors in BEAUti v1.10.4 except for a prior for the constant population size; we used a log-normal prior with a mean of 10 and standard deviation of 100 in real space. According to the results, we selected a strict clock model and constant size prior model for SC1-Japan cluster analysis. To obtain an effective sample size (ESS) greater than 200 for all

factors, we set the MCMC lengths to 1.0×10^7. We specified a general time-reversible substitution model with site rate heterogeneity modeled across four gamma distributions (GTR+Γ4) for all analyses.

We performed two phylogeographic analyses: one was an asymmetric discrete trait phylogeographic modeling for SC1 isolates using a Bayesian stochastic search variable selection framework and the other was a Bayesian structured coalescent approximation for SC1-Japan isolates using BASTA with an asymmetric migration model. For the asymmetric discrete trait phylogeographic modeling, we used all isolates in SC1 if five or more isolates were sampled from each country. We followed the same process as we describe above to prepare the input files.

We conducted a Bayesian structured coalescent approximation using BASTA (23), which was known to be more reliable under biased sampling conditions than discrete trait analysis, for SC1-Japan isolates to obtain supported traits of the strains.

This methodology was computationally expensive; therefore, we analyzed only isolates from Japan and the U.S. that were clustered into SC1-Japan by rhierBAPS. We clustered the tested isolates from Japan and the U.S. in SC1-Japan into three groups based on the branch lengths in Figure 2: the U.S. isolates in BASTA cluster 1, the U.S. isolates in BASTA cluster 2 and the Japanese isolates in BASTA cluster 2.

Supplementary Tables

Table S1. Strain information and PBP types of the tested serotype 19A-ST320 isolates recovered in Japan.

ID	Accession No.	Serotype	Sequence type	Year	Region	PBP type		
						pbp1a	*pbp2b*	*pbp2x*
ASP1347	DRR277768	19A	320	2018	Okinawa	13	11	16
ASP1356	DRR277769	19A	320	2018	Okinawa	13	11	16
ASP1516	DRR277770	19A	320	2018	Okinawa	13	11	16
ASP1756	DRR277771	19A	320	2019	Okinawa	13	11	16
ASP1994	DRR277772	19A	320	2019	Okinawa	13	11	16
ASP2069	DRR277773	19A	320	2020	Hokkaido	13	11	16
ASP678	DRR277774	19A	320	2016	Okinawa	13	11	16
KSP1076	DRR277775	19A	320	2014	Okinawa	13	11	16
KSP1078	DRR277776	19A	320	2014	Okinawa	13	11	16
KSP1081	DRR277777	19A	320	2014	Okinawa	13	11	16
KSP1106	DRR277778	19A	320	2014	Okinawa	13	11	16
KSP1156	DRR277779	19A	320	2014	Okinawa	13	11	16
KSP1157	DRR277780	19A	320	2014	Okinawa	13	11	16
KSP1195	DRR277781	19A	320	2014	Okinawa	13	11	16
KSP1321	DRR277782	19A	320	2014	Okinawa	13	11	16
KSP1405	DRR277783	19A	320	2015	Okinawa	13	11	16
KSP1406	DRR277784	19A	320	2015	Okinawa	13	11	16
KSP1536	DRR277785	19A	320	2016	Okinawa	13	11	JP60

KSP1642	DRR277786	19A	320	2017	Okinawa	13	11	16
KSP186	DRR277787	19A	320	2009	Okinawa	13	11	16
KSP338	DRR277788	19A	320	2010	Okinawa	13	11	JP61
KSP345	DRR277789	19A	320	2010	Okinawa	13	11	16
KSP349	DRR277790	19A	320	2010	Okinawa	13	11	16
KSP409	DRR277791	19A	320	2010	Fukuoka	13	11	16
KSP488	DRR277792	19A	320	2010	Okinawa	13	11	16
KSP504	DRR277793	19A	320	2011	Okinawa	13	11	16
KSP566	DRR277794	19A	320	2011	Okinawa	13	11	16
KSP594	DRR277795	19A	320	2011	Okinawa	13	11	16
KSP690	DRR277796	19A	320	2011	Okinawa	13	11	16
KSP727	DRR277797	19A	320	2012	Okinawa	13	11	16
KSP740	DRR277798	19A	320	2012	Okinawa	13	11	16
KSP748	DRR277799	19A	320	2012	Okinawa	13	11	16
KSP800	DRR277800	19A	320	2012	Okinawa	13	11	16
KSP801	DRR277801	19A	320	2012	Okinawa	13	11	16
KSP802	DRR277802	19A	320	2012	Okinawa	13	11	16
KSP803	DRR277803	19A	320	2012	Okinawa	13	11	16
KSP815	DRR277804	19A	320	2012	Okinawa	13	11	16
KSP831	DRR277805	19A	320	2013	Okinawa	13	11	16
KSP832	DRR277806	19A	320	2013	Okinawa	13	11	16
KSP840	DRR277807	19A	320	2013	Okinawa	13	11	16
KSP865	DRR277808	19A	320	2013	Okinawa	13	11	JP62

KSP867	DRR277809	19A	320	2013	Okinawa	13	11	16
KSP893	DRR277810	19A	320	2013	Okinawa	13	11	16
KSP974	DRR277811	19A	320	2013	Okinawa	13	11	16
KSP975	DRR277812	19A	320	2013	Okinawa	13	11	16
KSP988	DRR277813	19A	320	2013	Okinawa	13	11	16
SP1030	DRR277815	19A	320	2010	Tochigi	13	11	16
SP1236	DRR277816	19A	320	2011	Okinawa	13	11	16
SP220	DRR277817	19A	320	2009	Niigata	13	11	16
SP2662	DRR277818	19A	320	2013	Kanagawa	13	11	JP63
SP2674	DRR277819	19A	320	2013	Kanagawa	13	11	JP63
PC0297	DRR098639	19A	320	2013	Kumamoto	13	11	16
PC0396	DRR098652	19A	320	2013	Gifu	13	11	16
PC0576	DRR098668	19A	320	2014	Osaka	13	11	16
PC0641	DRR098675	19A	320	2014	Miyazaki	13	11	16
PC1521	DRR277814	19A	320	2017	Gifu	13	11	16

Table S2. Mapping coverage and genetic characteristics of the isolates from Japan tested in this study.

Isolate name	N_{50}	N_{75}	Coverage	*ermB*	*ermTR*	*mef*	*tetM*	*tetO*	Pili1	Pili2	*folA* I100L	*folA* D92R	*folP* insertion
ASP1347	60120	32676	209.8	yes	no	*mefE*	yes	no	yes	yes	yes	no	yes
ASP1356	67872	34530	142.8	yes	no	*mefE*	yes	no	yes	yes	yes	no	yes
ASP1516	84293	49138	82.7	yes	no	*mefE*	yes	no	yes	yes	yes	no	yes
ASP1756	68460	39220	196.6	yes	no	*mefE*	yes	no	yes	yes	yes	no	yes
ASP1994	75032	34231	215.1	yes	no	*mefE*	yes	no	yes	yes	yes	no	yes
ASP2069	67872	39045	234.6	yes	no	*mefE*	yes	no	yes	yes	yes	no	yes
ASP678	66795	32415	210.4	yes	no	*mefE*	yes	no	yes	yes	yes	no	yes
KSP1076	67925	33938	244.0	yes	no	*mefE*	yes	no	yes	yes	yes	no	yes
KSP1078	63595	34392	188.3	yes	no	*mefE*	yes	no	yes	yes	yes	no	yes
KSP1081	68613	34231	191.4	yes	no	*mefE*	yes	no	yes	yes	yes	no	yes
KSP1106	121124	52420	156.5	yes	no	*mefE*	yes	no	yes	yes	yes	no	yes
KSP1156	60916	36538	115.0	yes	no	*mefE*	yes	no	yes	yes	yes	no	yes
KSP1157	66734	28066	185.1	yes	no	*mefE*	yes	no	yes	yes	yes	no	yes
KSP1195	59723	32676	219.6	yes	no	*mefE*	yes	no	yes	yes	yes	no	yes
KSP1321	60784	32676	222.7	yes	no	*mefE*	yes	no	yes	yes	yes	no	yes
KSP1405	61010	32286	268.6	yes	no	*mefE*	yes	no	yes	yes	yes	no	yes
KSP1406	59266	28718	290.9	yes	no	*mefE*	yes	no	yes	yes	yes	no	yes
KSP1536	60688	32066	207.6	yes	no	*mefE*	yes	no	yes	yes	yes	no	yes
KSP1642	67872	32770	241.8	yes	no	*mefE*	yes	no	yes	yes	yes	no	yes

KSP186	68052	39275	217.2	yes	no	*mefE*	yes	no	yes	yes	yes	no	yes
KSP338	67926	34588	254.9	yes	no	*mefE*	yes	no	yes	yes	yes	no	yes
KSP345	66250	36971	272.5	yes	no	*mefE*	yes	no	yes	yes	yes	no	yes
KSP349	62667	33938	241.8	yes	no	*mefE*	yes	no	yes	yes	yes	no	yes
KSP409	67627	39284	263.3	yes	no	*mefE*	yes	no	yes	yes	yes	no	yes
KSP488	84966	49138	236.3	yes	no	*mefE*	yes	no	yes	yes	yes	no	yes
KSP504	68460	47946	230.4	yes	no	*mefE*	yes	no	yes	yes	yes	no	yes
KSP566	67595	52419	235.8	yes	no	*mefE*	yes	no	yes	yes	yes	no	yes
KSP594	59266	32286	298.7	yes	no	*mefE*	yes	no	yes	yes	yes	no	yes
KSP690	84956	35326	266.3	yes	no	*mefE*	yes	no	yes	yes	yes	no	yes
KSP727	76741	55805	274.1	yes	no	*mefE*	yes	no	yes	yes	yes	no	yes
KSP740	67595	39275	303.4	yes	no	*mefE*	yes	no	yes	yes	yes	no	yes
KSP748	59034	32529	245.7	yes	no	*mefE*	yes	no	yes	yes	yes	no	yes
KSP800	66543	34231	165.7	yes	no	*mefE*	yes	no	yes	yes	yes	no	yes
KSP801	58164	32286	221.5	yes	no	*mefE*	yes	no	yes	yes	yes	no	yes
KSP802	66271	32286	212.1	yes	no	*mefE*	yes	no	yes	yes	yes	no	yes
KSP803	63595	33938	196.5	yes	no	*mefE*	yes	no	yes	yes	yes	no	yes
KSP815	59711	33938	209.3	yes	no	*mefE*	yes	no	yes	yes	yes	no	yes
KSP831	68941	49138	218.2	yes	no	*mefE*	yes	no	yes	yes	yes	no	yes
KSP832	63112	33938	248.3	yes	no	*mefE*	yes	no	yes	yes	yes	no	yes
KSP840	67872	34392	270.3	yes	no	*mefE*	yes	no	yes	yes	yes	no	yes
KSP865	66808	33938	245.8	yes	no	*mefE*	yes	no	yes	yes	yes	no	yes
KSP867	76741	35632	260.8	yes	no	*mefE*	yes	no	yes	yes	yes	no	yes

KSP893	96590	56423	346.2	yes	no	*mefE*	yes	no	yes	yes	yes	no	yes
KSP974	90481	47649	238.4	yes	no	*mefE*	yes	no	yes	yes	yes	no	yes
KSP975	76741	39223	212.3	yes	no	*mefE*	yes	no	yes	yes	yes	no	yes
KSP988	83040	35223	280.0	yes	no	*mefE*	yes	no	yes	yes	yes	no	yes
SP1030	71027	52027	278.1	yes	no	*mefE*	yes	no	yes	yes	yes	no	yes
SP1236	68460	52420	235.2	yes	no	*mefE*	yes	no	yes	yes	yes	no	yes
SP220	87587	56401	209.2	yes	no	*mefE*	yes	no	yes	yes	yes	no	yes
SP2662	77177	39224	244.9	yes	no	*mefE*	yes	no	yes	yes	yes	no	yes
SP2674	77177	49138	262.0	yes	no	*mefE*	yes	no	yes	yes	yes	no	yes
PC0297	59722	36294	46.8	yes	no	*mefE*	yes	no	yes	yes	yes	no	yes
PC0396	72557	39045	52.6	yes	no	*mefE*	yes	no	yes	yes	yes	no	yes
PC0576	55847	29562	75.4	yes	no	*mefE*	yes	no	yes	yes	yes	no	yes
PC0641	57470	29365	59.4	yes	no	*mefE*	yes	no	yes	yes	yes	no	yes
PC1521	66436	34392	141.7	yes	no	*mefE*	yes	no	yes	yes	yes	no	yes

Table S3. Antimicrobial susceptibilities of serotype 19A-ST320 isolates from Japan*

	MIC (μg/ml)						
Isolate name	PCG	CTX	CEP	MEM	EM	ST	TC
ASP1347	4	2	2	1	>128	>76/4	32
ASP1356	4	4	2	1	>128	>76/4	32
ASP1516	4	2	2	1	>128	>76/4	32
ASP1756	8	2	2	1	>128	>76/4	32
ASP1994	4	2	2	1	>128	>76/4	32
ASP2069	4	2	2	1	>128	>76/4	32
ASP678	4	4	2	1	>128	>76/4	32
KSP1076	4	2	2	1	>128	>76/4	32
KSP1078	4	2	2	1	>128	>76/4	16
KSP1081	4	2	2	1	>128	>76/4	32
KSP1106	4	2	2	1	4	>76/4	32
KSP1156	4	2	2	1	>128	>76/4	32
KSP1157	4	2	2	1	>128	>76/4	32
KSP1195	4	2	2	1	>128	>76/4	32
KSP1321	4	2	2	1	>128	>76/4	32
KSP1405	4	2	2	1	>128	>76/4	32
KSP1406	4	2	2	1	>128	>76/4	32
KSP1536	8	>8	8	1	>128	>76/4	32
KSP1642	4	4	2	1	>128	>76/4	32
KSP186	4	2	2	1	>128	>76/4	32

KSP338	4	2	2	1	>128	>76/4	32
KSP345	4	2	2	1	>128	>76/4	32
KSP349	4	2	2	1	>128	>76/4	32
KSP409	4	2	2	1	>128	>76/4	32
KSP488	4	2	2	1	>128	>76/4	32
KSP504	8	2	2	1	>128	>76/4	32
KSP566	4	2	2	1	>128	>76/4	32
KSP594	4	2	2	1	>128	>76/4	32
KSP690	4	2	2	1	4	>76/4	32
KSP727	4	2	2	1	>128	>76/4	32
KSP740	4	2	2	1	>128	>76/4	32
KSP748	4	2	2	1	>128	>76/4	32
KSP800	4	2	2	0.5	>128	>76/4	32
KSP801	4	2	2	0.5	>128	>76/4	32
KSP802	4	2	2	1	>128	>76/4	16
KSP803	4	2	2	0.5	>128	>76/4	16
KSP815	4	2	2	1	>128	>76/4	16
KSP831	4	2	2	1	>128	>76/4	32
KSP832	4	2	2	0.5	>128	>76/4	16
KSP840	4	2	2	1	>128	>76/4	32
KSP865	8	>8	>8	1	>128	>76/4	32
KSP867	4	2	2	1	>128	>76/4	32
KSP893	4	2	2	1	>128	>76/4	32

KSP974	4	2	2	1	>128	>76/4	16
KSP975	4	2	2	1	>128	>76/4	32
KSP988	4	2	2	0.5	>128	>76/4	32
SP1030	4	2	2	1	>128	>76/4	32
SP1236	4	2	2	1	>128	>76/4	32
SP220	4	2	2	1	>128	>76/4	32
SP2662	8	>8	8	1	>128	>76/4	32
SP2674	4	>8	8	1	>128	>76/4	32
PC0297	4	2	2	0.5	>128	>76/4	32
PC0396	4	2	2	1	>128	>76/4	32
PC0576	4	2	2	1	>128	>76/4	32
PC0641	4	2	2	1	>128	>76/4	32
PC1521	4	2	2	1	>128	>76/4	32

*PCG, penicillin; CTX, cefotaxime; CEP, cefepime; MEM, meropenem; EM, erythromycin; ST, trimethoprim-sulfamethoxazole; TC, tetracycline.

Table S4. Recombination site information in each sequence cluster (SC) detected by Gubbins.

SC	Minimum length	1st Quartile	Median	Mean	3rd Quartile	Maximum length	No. of recombination sites
SC1	5	146	1207	4261	5457	77450	2080
SC2	5	261	1379	4639	5192	69904	1290
SC3	5	732	2706	6503	8323	96555	622
SC4	5	127	654	4251	3130	36502	101
SC5	15	313	948	5358	5652	75163	175
SC6	NA	NA	NA	NA	NA	NA	NA
SC7	5	131	683	4888	4621	61846	186
SC8	5	60	437	3537	2880	67719	338
SC9	5	60	264	2298	2077	45276	249
SC10	17	528	3535	5424	8414	35096	333
SC11	7	228	1933	4811	6909	76857	505
SC12	NA	NA	NA	NA	NA	NA	NA
SC13	NA	NA	NA	NA	NA	NA	NA
SC14	NA	NA	NA	NA	NA	NA	NA
SC15	23	309	2487	6788	8661	56761	142
SC16	NA	NA	NA	NA	NA	NA	NA
SC17	9	595	4617	6915	10727	42626	294
SC18	NA	NA	NA	NA	NA	NA	NA
SC19	NA	NA	NA	NA	NA	NA	NA
SC20	NA	NA	NA	NA	NA	NA	NA
SC_all	5	219	1403	4967	6014	96555	6997

Supplementary data

pbp1a
>JP_1a1
SMKPITDYAPALEYGVYDSTASIVHDVPYNYPGTDTPLYNWDHVYFGNITIQYALQQSRNVTAVETLNKVGLDRAKTFLNGL
GIDYPSMHYANAISSNTTESNKKYGASSEKMAAAYAAFANGGIYHKPMYINKIVFSDGSEKEFSDAGTRAMKETTAYMMTEM
MKTVLTYGTGRGAYLPWLPQAGKTGTSNYTDEEIEKYIKNTGYVAPDEMFVGYTRKYSMAVWTGYSNRLTPIVGDGFLVAA
KVYRSMITYLSEDTHPEDWTMPDGLFRNGEFV
>JP_1a2
SMKPITDYAPALEYGVYDSTATIVHDEPYNYPGTDIPVYNWDRGYFGNITLQYALQQSRNVPAVETLNKVGLNRAKTFLNGL
GIDYPSLHYSNAISSNTTESDQKYGASSEKMAAAYAAFANGGTYYKPMYIHKVVFSDGSEKEFSNVGTRAMKETTAYMMTD
MMKTVLTYGTGRGAYLPWLPQAGKTGTSNYTDEEIEKYIKNTGYVAPDEMFVGYTRKYSMAVWTGYSNRLTPLVGNGLTV
AAKVYRSMMTYLSEGSNPEDWNIPEGLYRNGEFV
>JP_1a3
SMKPITDYAPALEYGVYDSTASIVHDVPYNYPGTDTPLYNWDHVYFGNITIQYALQQSRNVTAVETLNKVGLDRAKTFLNGL
GIDYPSMHYANAISSNTTESNKKYGASSEKMAAAYAAFANGGIYHKPMYINKIVFSDGSEKEFSDAGTRAMKETTAYMMTD
MMKTVLSYGTGRNAYLAWLPQAGKTGTSNYTDEEIEKYIKNTGYVAPDEMFVGYTRKYSMAVWTGYSNRLTPIVGDGFLVA
AKVYRSMMTYLSEGSNPEDWNIPEGLYRNGEFV
>JP_1a4
TMKPITDYAPAIEYGIYDSTATMVNDIPYNYPGTSTPVYNWDRAYFGNITLQYALQQSRNVPAVETLNKVGLDRAKNFLNGLG
IDYPDMHYSNAISSNTTESNKQYGASSEKMAAAFAAFANGGIYHKPMYINKIVFSDGSEKEFSDAGTRAMKETTAYMMTEMM
KTVLSYGTGRNAYLAWLPQAGKTGTSNYTDEEIENHIKTSQFVAPDELFAGYTRKYSMAVWTGYSNRLTPLVGNGLTVAAK
VYRSMMTYLSEGSNPEDWNIPEGLYRNGEFV
>JP_1a5
TMKPITDYAPAIEYGVYDSTATMVNDIPYNYPGTSTPVYNWDRAYFGNITLQYALQQSRNVPAVETLNKVGLDRAKNFLNGL
GIDYPDMHYSNAISSNTTESNKQYGASSEKMAAAFAAFANGGIYHKPMYINKIVFSDGSEKEFSDAGTRAMKETTAYMMTEM
MKTVLSYGTGRNAYLAWLPQAGKTGTSNYTDEEIENHIKTSQFVAPDELFVGYTRKYSMAVWTGYSNRLTPLVGNGLTVAA
KVYRSMMTYLSEGSNPEDWNIPEGLYRNGEFV
>JP_1a6
SMKPITDYAPALEYGVYDSTASIVHDVPYNYPGTDTPLYNWDHVYFGNITIQYALQQSRNVTAVETLNKVGLDRAKTFLNGL

GIDYPSMHYANAISSNTTESNKKYGASSEKMAAAYAAFANGGIYHKPMYIHKVVFSDGSEKEFSDAGTRAMKETTAYMMTE
MMKTVLTYGTGRGAYLPWLPQAGKTGTSNYTDEEIEKYIKNTGYVAPDEMFVGYTRKYSMAVWTGYSNRLTPIVGDGFLVA
AKVYRSMMTYLSEGSNPEDWNIPEGLYRNGEFV
>JP_1a7
TMKPITDYAPAIEYGVYDSTATMVNDIPYNYPGTSTPVYNWDRAYFGNITLQYALQQSRNVPAVETLNKVGLDRAKNFLNGL
GIDYPDMHYSNAISSNTTESNKQYGASSEKMAAAFAAFANGGIYHKPMYINKIVFSDGSEKEFSDAGTRAMKETTAYMMTEM
MKTVLSYGTGRNAYLAWLPQAGKTGTSNYTDKEIENHIKTSQFVAPDELFAGYTRKYSMAVWTGYSNRLTPLVGNGLTVAA
KVYRSMMTYLSEGSNPEDWNIPEGLYRNGEFV
>JP_1a8
SMKPITDYAPALEYGVYDSTATMVNDIPYNYPGTSTPVYNWDRAYFGNISLQYALQQSRNVPAVETLNKVGLDRAKTFLNGL
GIDYPSIHYSNAISSNTTESSKQYGASSEKMAAAYAAFANGGIYHKPMYINKVVFSDGSEKEFSDAGTRAMKETTAYMMTEM
MKTVLTYGTGRGAYLPWLPQAGKTGTSNYTDDEIEKYVKNTGYVAPDEMFVGYTRKYSMAVWTGYSNRLTPIIGDGFLVAA
KVYRSMISYLSEDDHPGDWTMPEGVYRSGEFV
>JP_1a9
SMKPITDYAPALEYGVYDSTATMVNDIPYNYPGTSTPVYNWDRAYFGNISLQYALQQSRNVPAVETLNKVGLDRAKTFLNGL
GIDYPSIHYSNAISSNTTESSKQYGASSEKMAAAYAAFANGGIYHKPMYINKVVFSDGSEKEFSDTGTRAMKETTAYMMTEM
MKTVLTYGTGRGAYLPWLPQAGKTGTSNYTDDEIEKYIKNTGYVAPDEMFVGYTRKYSMAVWTGYSNRLTPIIGDGFLVAA
KVYRSMISYLSEDDHPGDWTMPEGVYRSGEFV
>JP_1a10
SMKPITDYAPALEYGVYDSTASIVHDVPYNYPGTDTPLYNWDHVYFGNITIQYALQQSRNVTAVETLNKVGLDKAKTFLNGL
GIDYPSMHYANAISSNTTESNKKYGASSEKMAAAYAAFANGGIYHKPMYINKIVFSDGSEKEFSDAGTRAMKETTAYMMTEM
MKTVLTYGTGRGAYLPWLPQAGKTGTSNYTDEEIEKYIKNTGYVAPDEMFVGYTRKYSMAVWTGYSNRLTPIVGDGFLVAA
KVYRSMITYLSEDTHPEDWTMPDGLFRNGEFV
>JP_1a11
TMKPITDYAPALEYGVYDSTATIVHDEPYNYPGTNTPVYNWDRGYFGNITLQYALQQSRNVPAVETLNKVGLNRAKTFLNGL
GIDYPSIHYSNAISSNTTESDKKYGASSEKMAAAYAAFANGGTYYKPMYIHKVVFSDGSEKEFSNVGTRAMKETTAYMMTDM
MKTVLSYGTGRNAYLAWLPQAGKTGTSNYTDEEIENHIKTSQFVAPDELFAGYTRKYSMAVWTGYSNRLTPLVGNGLTVAA
KVYRSMMTYLSEGGNPEDWNIPEGLYRNGEFV
>JP_1a12
TMKPITDYAPALEYGVYDSTATIVHDEPYNYPGTNTPVYNWDRGYFGNITLQYALQQSRNVPAVETLNKVGLNRAKTFLNGL

GIDYPSIHYSNAISSNTTESDKKYGASSEKMAAAYAAFANGGTYYKPMYIHKVVFSDGSEKEFSNVGTRAMKKTTAYMMTD
MMKTVLSYGTGRNAYLAWLPQAGKTGTSNYTDEEIENHIKTSQFVAPDELFAGYTRKYSMAVWTGYSNRLTPLVGNGLTVA
AKVYRSMMTYLSEGSNPEDWNIPEGLYRNGEFV
>JP_1a13
TMKPITDYAPALEYGVYDSTATIVHDEPYNYPGTNTPVYNWDRGYFGNITLQYALQQSRNVPAVETLNKVGLNRAKTFLNGL
RIDYPSIHYSNAISSNTTESDKKYGASSEKMAAAYAAFANGGTYYKPMYIHKVVFSDGSEKEFSNVGTRAMKETTAYMMTDM
MKTVLSYGTGRNAYLAWLPQAGKTGTSNYTDEEIENHIKTSQFVAPDELFAGYTRKYSMAVWTGYSNRLTPLVGNGLTVAA
KVYRSMMTYLSEGSNPEDWNIPEGLYRNGEFV
>JP_1a14
TMKPITDYAPALEYGVYDSTATIVHDEPYNYPGTNTPVYNWDRGYFGNITLQYALQQSRNVPAVETLNKVGLNRAKTFLNGL
GIDYPSIHYSNAISSNTTESDKKYGASSEKMAAAYAAFANGGTYYKPMYIHKVVFSDGSEKEFSNVGTRAMKETTTYMMTDM
MKTVLTYGTGRNAYLAWLPQAGKTGTSNYTDEEIENHIKTSQFVAPDELFAGYTRKYSMAVWTGYSNRLTPLVGNGLTVAA
KVYRSMMTYLSEGSNPEDWNIPEGLYRNGEFV
>JP_1a15
SMKPITDYAPALEYGVYDSTASIVHDVPYNYPGTDTPLYNWDHVYFGNITIQYALQQSRNVTAVETLNKVGLDRAKTFLNGL
GIDYPSMHYANAISSNTTESNKKYGASSEKMAAAYAAFANGGIYHKPMYINKIVFSDGSEKEFSDAGTRAMKETTAYMMTEM
MKTVLTYGTGRGAYLPWLPQAGKTGTSNYTDEEIEKYIKNTGYVAPDEMFVGYTRKYSMAVWTGYSNRLTPIVGDGFLVAA
KVYRSMISYLSEDDHPGDWTMPEGLYRSGEFV
>JP_1a16
SMKPITDYAPALEYGVYDSTASIVHDVPYNYPGTDTPLYNWDHVYFGNITIQYALQQSRNVTAVETLNKVGLDRAKTFLNGIG
IDYPDMHYANAISSNTTESNKKYGASSEKMAAAYAAFANGGIYHKPMYINKIVFSDGSSKEYADPGTRAMKETTAYMMTEM
MKTVLAYGTGRGAYLPWLPQAGKTGTSNYTDDEIENYIKNTGYVAPDEMFVGYTRKYSMAVWTGYSNRLTPIVGDGFYVA
AKVYRSMMTYLSEDNNPGDWTMPDGLFRNGEFV
>JP_1a17
SMKPITDYAPALEYGVYDSTATIVHDEPYNYPGTDIPVYNWDRGYFGNITLQYALQQSRNVPAVETLNKVGLNRAKTFLNGL
GIDYPSLHYSNAISSNTTESDQKYGASSEKMAAAYAAFANGGTYYKPMYIHKVVFSDGSEKEFSNVGTRAMKETTAYMMTD
MMKTVLTYGTGRGAYLPWLPQAGKTGTSNYTDEEIEKYIKNTGYVAPDEMFVGYTRKYSMAVWTGYSNRLTPIIGDGFLVA
AKVYRSMMTYLSEGSNPEDWNIPEGLYRNGEFV
>JP_1a18
SMKPITDYAPALEYGVYDSTATIVHDEPYNYPGTDIPVYNWDRGYFGNITLQYALQQSRNVPAVETLNKVGLNRAKTFLNGL

GIDYPSLHYSNAISSNTTESDQKYGASSEKMAAAYAAFANGGTYYKPMYIHKVVFSDGSEKEFSNVGTRAMKETTAYMMTD
MMKTVLTYGTGRGAYLPWLPQAGKTGTSNYTDEEIEKYIKNTGYVAPDEMFVGYTRKYSMAVWTGYSNRLTPIIGDGFLVA
AKVYRSMMTYLSEGSNPEDWNMPDGIYRNGEFV

pbp2b

>JP_2b1
TNVFVPGSVVKAATISSGWENGVLSGNQTLTDQPIVFQGSAPIYSWYKLAYGSFPITAVLEYSSNAYMVQTALGIMGQTYQPN
MFVGTSNLESAMEKLRSTFGEYGLGTATGIDLPDTGFVPKEYSFANYITNAFGQFDNYTPMQLAQYVATIANNGVRVAPRIVE
GIYGNNDKGGDLIQQLQPTEMNKVNISDSDMSILHQGFYQVAHGTSGLTTGRAFSNGAAVSISGKTGESYVEGGQEANNTNA
VAYAPSDNPQIAVAVVFPHNTN
>JP_2b2
TNVFVPGSVVKAATISSGWENGVLSGNQTLTDQPIVFQGSAPIYSWYKLAYGSFPITAVLEYSSNAYMVQTALGIMGQTYQPN
MFVGTSNLETAMGKLRATFGEYGLGAATGIDLPDTGFVPKEYSFANFITNAFGQFDNYTPMQLAQYVATIANNGVRLAPHIVE
GIYDNNDKGGELIQAIDTKEINKVNISESDMAILHQGFYQVSHGTSPLTTGRAFSDGATVSISGKTGESYVAGGQEANNTNAVA
YAPTENPQIAVAVVFPHNTN
>JP_2b3
TNVFAPGSVVKAATISSGWENGVLSGNQTLTDQSIVFQGSAPINSWYTQAYGSFPITAVLEYSSNAYMVQTALGLMGQTYQPN
MFVGTSNLESAMGKLRSTFGEYGLGSATGIDLPDTGFVPKDYSFANYITNAFGQFDNYTPMQLAQYVATIANDGVRVAPRIVE
GIYGNNDKGGDLIQQLQPTEMNKVNISDSDMSILHQGFYQVAHGTSGLTTGRAFSNGAAVSISGKTGESYVEGGQEANNTNA
VAYAPSDNPQIAVAVVFPHNTN
>JP_2b4
TNVFVPGSVVKAATISSGWENGVLSGNQTLTDQSIVFQGSAPINSWYTQAYGSFPITAVLEYSSNAYMVQTALGLMGQTYQPN
MFVGTSNLESAMGKLRSTFGEYGLGSATGIDLPDTGFVPKDYSFANYITNAFGQFDNYTPMQLAQYVATIANDGVRVAPRIVE
GIYGNNDKGGDLIQQLQPTEMNKVNVSDSDMSILHQGFYQVAHGTSGLTTGRAFSNGAAVSISGKTGESYVEGGQEANNTNA
VAYAPSDNPQIAVAVVFPHNTN
>JP_2b5
TNVFVPGSVVKAATISSGWENGVLSGNQTLTDQSIVFQGSAPINSWYTQAYGSFPITAVLEYSSNVYMVQTALGLMGQTYQPN
MFVGTSNLESAMGKLRSTFGEYGLGSATGIDLPDTGFVPKDYSFANYITNAFGQFDNYTPMQLAQYVATIANDGVRVAPRIVE
GIYGNNDKGGDLIQQLQPTEMNKVNISDSDMSILHQGFYQVAHGTSGLTTGRAFSNGAAVSISGKTGESYVEGGQEANNTNA
VAYAPSDNPQIAVAVVFPHNTN
>JP_2b6
TNVFVPGSVVKAATISSGWENGVLSGNQTLTDQSIVFQGSAPINSWYTQAYGSFPITAVLEYSSNTYMVQTALGLMGQTYQPN
MFVGTSNLESAMEKLRSTFGEYGFGTATGIDLPDIGFVPKEYSFANYITNAFGQFDNYTPMQLAQYVATIANNGVRVAPRIVEG
IYGNNDKGGDLIQQLQPTEMNKVNISDSDMSILHQGFYQVAHGTSGLTTGRAFSNGALVSISGKTGESYVADGQQATNTNAV

AYAPSDNPQIAVAVVFPHNTN
>JP_2b7
TNVFVPGSVVKAATISSGWENGVLSGNQTLTDQSIVFQGSAPINSWYTQAYGSFPITAVLEYSSNTYMVQTALGLMGQTYQPN
MFVGTSNLESAMEKLRSTFGEYGLGTATGIDLPDIGFVPKEYSFANYITNAFGQFDNYTPMQLAQYVATIANNGVRVAPRIVE
GIYGNNDKGGDLIQQLQPTEMNKVNISDSDMSILHQGFYQVAHGTSGLTTGRAFSNGALVSISGKTGESYVADGQQATNTNA
VAYAPSDNPQIAVAVVFPHNTN
>JP_2b8
TNVFVPGSVVKAATISSGWENGVLSGNQTLTDQPIVFQGSAPIYSWYKLAYGSFPITAVLEYSSNAYMVQTALGIMGQTYQPN
MFVGTSNLETAMGKLRATFGEYGFGAATGIDLPDTGFVPKEYSFANYITNAFGQFDNYTPMQLAQYVATIANDGVRVAPRIVE
GIYGNNDKGGDLIQQLQPTEMNKVNISDSDMSILHQGFYQVAHGTSGLTTGRAFSNGALVSISGKTGESYVADGQQATNTNA
VAYAPSDNPQIAVAVVFPHNTN
>JP_2b9
TNVFVPGSVVKAATISSGWENGVLSGNQTLTDQPIVFQGSAPIYSWYKLAYGSFPITTVLEYSSNAYMVQTALGIMGQTYQPN
MFVGTSNLETAMGKLRATFGEYGLGAATGIDLPDTGFVPKEYSFANYITNAFGQFDNYTPMQLAQYVATIANDGVRVAPRIV
EGIYGNNDKGGDLIQQLQPTEMNKVNISDSDMSILHQGFYQVAHGTSGLTTGRAFSNGALVSISGKTGESYVAGGQEANNTNA
VAYAPSDNPQIAVAVVFPHNTN
>JP_2b10
TNVFVPGSVVKAATISSGWENGVLSGNQTLTDQPIVFQGSAPIYSWYKLAYGSFPITAVLEYSSNAYMVQTALGIMGQTYQPN
MFVGTSNLETAMGKLRATFGEYGLGAATGIDLPDTGFVPKEYSFANYITNAFGQFDNYTPMQFAQYVATIANDGVRVAPRIVE
GIYGNNDKGGDLIQQLQPTEMNKVNISDSDMSILHQGFYQVAHGTSGLTTGRAFSNGALVSISGKTGESYVAGGQEANNTNA
VAYAPSDNPQIAVAVVFPHNTN
>JP_2b11
TNVFVPGSVVKAATISSGWENGVLSGNQTLTDQPIVFQGSAPIYSWYKLAYGSFPITAVLEYSSNAYMVQTALGIMGQTYQPN
MFVGTSNLETAMGKLRATFGEYGLGAATGIDLPDTGFVPKEYSFANYITNAFGQFDNYTPMQLAQYVATIANDGVRVAPRIV
EGIYGNNDKGGDLIQQLQPTEMNKVNISDSDMSILHQGFYQVAHGTSGLTTGRAFSNGALVSISGKTGESYVAGSQEANNTNA
VAYAPSDNPQIAVAVVFPHNTN
>JP_2b12
TNVFVPGSVVKAATISSGWENGVLSGNQTLTDQPIVFQGSAPIYSWYKLAYGSFPITAVLEYSSNAYMVQTALGIMGQTYQPN
MFVGTSNLETAMGKLRATFGEYGLGVATGIDLPDTGFVPKEYSFANYITNAFGQFDNYTPMQLAQYVATIANDGVRVAPRIV
EGIYGNNDKGGELIQAIDTKEINKVNISESDMAILHQGFYQVSHGTSPLTTGRAFSDGATVSISGKTGESYVAGGQEANNTNAV

AYAPTENPQIAVAVVFPHNTN
>JP_2b13
TNVFVPGSVVKAATISSGWENGVLSGNQTLTDRSIVFQGSAPINSWYTQAYGSFPITAVLEYSSNAYMVQTALGLMGQTYQPN
MFVGTSNLESAMGKLRSTFGEYGLGSATGIDLPDTGFIPKDYSFANYITNAFGQFDNYTPMQLAQYVATIANDGVRVAPRIVE
GIYGNNDKGGDLIQQLQPTEMNKVNISDSDMSVLHQGFYQVAHGTSGLTTGRAFSNGASVSISGKTGESYVEGGQEANNTNA
VAYAPSDNPQIAVAVVFPHNTN
>JP_2b14
TNVFVPGSVVKAATISSGWENGVLSGNQTLTDQSIVFQGSAPINSWYTQAYGSFPITAVLEYSSNAYMVQTALGIMGQTYQPN
MFVLTNNLESAMGKLRSTFAEYGLGASTGIDLPDTGFIPKEYNFANYITNAFGQFDNYTPMQLAQYVATIANDGVRVAPRIVE
GIYGNNDKGGDLIQQLQPTEMNKVNISDSDMSVLHQGFYQVAHGTSGLTTGRAFSNGALVSISGKTGESYVADGQQATNTNA
VAYAPSDNPQIAVAVVFPHNTN
>JP_2b15
TNVFVPGSVVKAATISSGWENGVLSGNQTLTDQPIVFQDSAPIYSWYKLAYGSFPITAVLEYSSNAYMVQTALGIMGQTYQPN
MFVGTSNLESAMEKLRSTFGEYGLGTATGIDLPDTGFVPKEYSFANYITNAFGQFDNYTPMQLAQYVATIANNGVRVAPRIVE
GIYGNNDKGGDLIQQLQPTEMNKVNISDSDMSILHQGFYQVAHGTSGLTTGRAFSNGAAVSISGKTGESYVEGGQEANNTNA
VAYAPSDNPQIAVAVVFPHNTN
>JP_2b16
TNVFVPGSVVKAATISSGWENGVLSGNQTLTDQPIVFQGSAPIYSWYKLAYGSFPITAVLEYSSNAYMVQTALGIMGQTYQPN
MFVGTSNLETAMGKLRATFGEYGLGAATGIDLPDTGFVPKEYSFANYITNAFGQFDNYTPMQLAQYVATIANNGVRVAPRIV
EGIYGNNDKGGDLIQQLQPTEMNKVNISDSDMSILHQGFYQVAHGTSGLTTGRAFSNGASVSISGKTGESYVEGGQEANNTNA
VAYAPSDNPQIAVAVVFPHNTN
>JP_2b17
TNVFVPGSVVKAATISSGWENGVLSGNQTLTDQSIVFQGSAPINSWYTQAYGSFPITAVLEYSSNTYMVQTALGLMGQTYQPN
MFVGTSNLESAMEKLRSTFGEYGLGTATGIDLPDTGFVPKEYSFANYITNAFGQFDNYTPMQLAQYVATIANDGVRVAPRIVE
GIYGNNDKGGDLIQQLQPTEINKVNISDSDMSILHQGFYQVAHGTSGLTTGRAFSNGALVSISGKTGESYVADGQQATNTNAV
AYAPSDNPQIAVAVVFPHNTN
>JP_2b18
TNVFVPGSVVKAATISSGWENGVLSGNQTLTDQSIFFQGSAPINSWYTQAYGSFPITAVLEYSSNTYMVQTALGLMGQTYQPN
MFVGTSNLESAMEKLRSTFGEYGLGTATGIDLPDTGFVPKEYSFANYITNAFGQFDNYTPMQLAQYVATIANNGVRVAPRIVE
GIYGNNDKGGDLIQQLQPTEMNKVNISDSDMSILHQGFYQVAHGTSGLTTGRAFSNGALVSISGKTGESYVADGQQATNTNA

VAYAPSDNPQIAVAVVFPHNTN
>JP_2b19
TNVFVPGSVVKAATISSGWENGVLSGNQTLTDQSIVFQGSAPINSWYTQAYGSFPITAVLEYSSNTYMVQTALGLMGQTYQPN
MFVGTSNLESAMEKLRSTFGEYGLGTATGIDLPDTGFVPKEYSFANYITNAFGQFDNYTPMQLAQYVATIANNGVRVAPRIVE
GIYGNNDKGGDLIQQLQPTEMNKVNISDSDMSILHQGFYQVAHGTSALTTGRAFSNGAAVSISGKTGESYVAGGQKADNTNA
VAYAPSDNPQIAVAVVFPHNTN
>JP_2b20
TNVFVPGSVVKAATISSGWENGVLSGNQTLTDQSIVFQGSAPINSWYTQAYGSFPITAVLEYSSNTYMVQTALGLMGQTYQPN
MFVGTSNLESAMEKLRSTFGEYGLGTATGIDLPDTGFVPKEYSFANYITNAFGQFDNYTPMQLAQYVATIANNGVRVAPRIVE
GIYGNNDKGGDLIQQLQPTEMNKVNISDSDMSILHQGFYQVAHGTSELTTGRAFLNGALVSISGKTGESYVADGQQATNTNA
VAYAPSDNPQIAVAVVFPHNTN
>JP_2b21
TNVFVPGSVVKAATISSGWENGVLSGNQTLTDQSIVFQGSAPINSWYTQAYGSFPITAVLEYSSNSYMVQTALGLMGQTYQPN
MFVGTSNLESAMEKLRSTFGEYGLGTATGIDLPDTGFVPKEYSFANYITNAFGQFDNYTPMQLAQYVATIANNGVRVAPRIVE
GIYGNNDKGGDLIQQLQPTEMNKVNISDSDMSILHQGFYQVAHGTSGLTTGRAFSNGALVSISGKTGESYVADGQQATNTNA
VAYAPSDNPQIAVAVVFPHNTN

pbp2x

>JP_2x1
GTDGIITYEKDRLGNIVPGTEQVSQQTVDGKDVYTTISSPLQSFMETQMDAFQEKVKGKTATLVSAKTGEILATTQRPTFDADT
KEGITEDFVWRDILYQSNYEPGSPMKVMMLAAANNTFPGGEVFNSSELKIADATIRDWDVNEGLTGGRMMTFSQGFAHSSNV
GMTLLEQKMATWLDYLNRFKFGVPTRFGLTDEYAGQLPADNIVNIAMSAFGQGISVTQTQMLRAFTANDGVMLEPKFISALY
DPNDQSVRKSQKEIVGNPVSKEAASVTRDHMVMVGTDPTYGTMHSTGKATVNVPGQNVALKSGTAEIADEKNGGYLTGSTN
NIFSVVSMHPAENPDFILYV
>JP_2x2
GKDGIITYEKDRLGNIVPGTEQVSQQTVDGKDVYTTISSTLQSFMETQMDAFLEKVKGKTATLVSAKTGEILATTQRPTFNADT
KEGITEDFVWRDILYQSNYEPGSAMKVMTLASSNNTFPSGEYFNSSEFKIADATTRDWDVNDGLTTGGMMTFLQGFAHSSNV
GMSLLEQKMATWLDYLSRFKFGVPTRFGLTDEYAGQLPADNIVSIAQSSFGQGISVTQTQMLRAFTANDGVMLEPKFISAIYDT
NNQSVRKSQKEIVGNPVSKEAASTTRNHMILVGTDPLYGTMHYTGKPIITVPGQNVAVKSGTAQIADEKNGGYLVGSTNYIFS
VVTMNPAENPDFILYV
>JP_2x3
GKDGIITYEKDRLGNIVPGTEQVSQQTVDGKDVYTTLSSPLQSFMETQMDAFLEKVKGKTATLVSAKTGEILATTQRPTFNAD
TKEGITEDFVWRDILYQSNYEPGSAFKVMMLASSNNTFPSGEYFNSSEFKIADATTRDWDVNEGLTTGGMMTFSQGFAHSSNV
GTSLLEQKMATWLDYLKRFKFGVPTRFGLTDEYAGQLPADNIVSIAQSSFGQGISVTQTQMLRAFTANDGVMLEPKFISAIYDT
NNQSVRKSQKEIVGNPVSKEAASTTRNHMILVGTDPLYGTMHYTGKPIITVPGQNVAVKSGTAQIADEKNGGYLVGSTNYIFS
VVTMNPAENPDFILYV
>JP_2x4
GTDGIITYEKDRVGNIVPGTELVSQQTVDGKDVYTTLSSPLQSFMETQMDAFLEKVKGKTATLVSAKTGEILATTQRPTFNADT
KEGITEDFVWRDILYQSNYEPGSAMKVMTLAASNNTFPSGEYFNSSEFKIADATTRDWDVNEGLTTGGMMTFLQGFAHSSNV
GMSLLEQKMATWLDYLKRFKFGVPTRFGLTDEYAGQLPADNIVSIAQSSFGQGISVTQTQMLRAFTANDGVMLEPKFISAIYD
TNNQSVRKSQKEIVGNPVSKEAASTTRNHMILVGTDPLYGTMHYTGKPIITVPGQNVAVKSGTAQIADEKNGGYLVGSTNYIF
SAVTMNPAENPDFILYV
>JP_2x5
GTDGIITYEKDRLGNIVPGTELVSQQTVDGKDVYTTLSSPLQSFMETQMDAFLEKVKGKTATLVSAKTGEILATTQRPTFNADT
KEGITEDFVWRDILYQSNYEPGSAMKVMTLASSNNTFPSGEYFNSSEFKIADATTRDWDVNEGLTTGGMMTFLQGFAHSSNV
GMSLLEQKMATWLDYLKRFKFGVPTRFGLTDEYAGQLPADNIVSIAQSSFGQGISVTQTQMLRAFTANDGVMLEPKFISAIYD
TNNQSVRKSQKEIVGNPVSKEAASTTRNHMILVGTDPLYGTMHYTGKPIITVPGQNVAVKSGTAQIADEKNGGYLVGSTNYIF

SAVTMNPAENPDFILYV
>JP_2x6
GKDGIITYEKDRLGNIVPGTEQVSQQTVDGKDVYTTLSSPLQSFMETQMDAFLEKVKGKTATLVSAKTGEILATTQRPTFNAD
TKEGITEDFVWRDILYQSNYEPGSAFKVMMLASSNNTFPSGEYFNSSEFKIADATTRDWDVNAGLTTGGMMTFLQGFVHSSN
VATSLLEQKMATWLDYLKRFKFGVPTRFGLTDEYAGQLPADNIVSIAQSSFGQGISVTQTQMLRAFTANDGVMLEPKFISAIYD
TNNQSVRKSQKEIVGNPVSKEAASTTRNHMILVGTDPLYGTMHYTGKPIITVPGQNVAVKSGTAQIADEKNGGYLVGSTNYIF
SVVTMNPAENPDFILYV
>JP_2x7
GTDGIITYEKDRLGNIVPGTEQVSQQTVDGKDVYTTLSSPLQSFMETQMDAFLEKVKGKTATLVSAKTGEILATTQRPTFNADT
KEGITEDFVWRDILYQSNYEPGSAMKVMTLASSNNTFPSGEYFNSSEFKIADATTRDWDVNAGLTTGGMMTFLQGFAHSSNV
GMSLLEQKMATWLDYLKRFKFGVPTRFGLTDEYAGQLPADNIVSIAQSSFGQGISVTQTQMLRAFTANDGVMLEPKFISAIYD
TNNQSVRKSQKEIVGNPVSKEAASTTRNHMILVGTDPIYGTMHYTGKPIITVPGQNVAVKSGTAQIADEKNGGYLVGSTNYIFS
VVTMNPAENPDFILYV
>JP_2x8
GKDGIITYEKDRLGNIVPGTEQVSQQTVDGKDVYTTISSTLQSFMETQMNAFQEKVKGKTATLVSAKTGEILATTQRPTFDADT
KEGLTKDFVWRDILYQSNYEPGSAMKVMTLAAANNTFPGGEVFNSSELKVADVTTRDWDVNEGLTGGGMMTFSQGFAHSS
NVGMTLLEQKMATWLDYLNRFKFGVPTRFGLTDEYAGQLPADNIVNIAQSSFGQGISVTQTQMIRAFTANDGVMLEPKFISAI
YDPNDQTARKSQKEIVGNPVSKDAASLTRTHMVLVGTDPVYGTMHKTGKPTVTVPGQNVALKSGTAQIADEKNGGYLVGLT
NYIFSAVSMNPAENPDFILYV
>JP_2x9
GKDGIITYEKDRLGNIVPGTEQVSQQTVDGKDVYTTISSTLQSFMETQMNVFQEKVKGKTATLVSAKTGEILATTQRPTFDADT
KEGLTKDFVWRDILYQSNYEPGSTMKVMTLAAANNTFPSGEYFNSSELKIADVTIRDWDVNDGLTTGRMMTFLQGFALSSNV
GMTLLEQKMATWLDYLNRFKFGVPTRFGLTDEYAGQLPADNIVNIAQSSFGQGISVTQTQMIRAFTANDGVMLEPKFISAIYD
PNDQTARKSQKEIVGNPVSKDAASLTRTNMVLVGTDPVYGTMHSTGKPTVTVPGQNVALKSGTAQIADEKNGGYLVGLTNYI
FSAVSMSPAENPDFILYV
>JP_2x10
GTDGIITYEKDRLGNIVPGTEQVSQQTVDGKDVYTTISSTLQSFMETQMNAFQEKVKGKTATLVSAKTGEILATTQRPTFDADT
KEGLTKDFVWRDILYQSNYEPGSTMKVMTLAAANNTFPGGEVFNSSELKIADVTIRDWDVNDGLTTGRMMTFLQGFALSSNV
GMSLLEQKMTTWLDYLNRFKFGVPTRFGLTDEYAGQLPADNIVNIAQSSFGQGISVTQTQMLRAFTANDGVMLEPKFISAIYD
PNDQTARKSQKEIVGNPVSKDAASLTRTNMVLVGTDPVYGTMHSTGKPTVTVPGQNVALKSGTAQIADEKNGGYLVGLTNYI

FSAVSMNPAENPDFILYV
>JP_2x11
GTDGIITYEKDRLGNIVPGTEQVSQQTVDGKDVYTTISSPLQSFMETQMDAFQEKVKGKTATLVSAKTGEILATTQRPTFDADT
KEGITEDFVWRDILYQSNYEPGSTMKVMMLAAANNTFPGGEVFNSSELKIADATIRDWDVNEGLTGGRMMTFSQGFAHSSNV
GMTLLEQKMATWLDYLNRFKFGVPTRFGLTDEYAGQLPADNIVNIAMSAFGQGISVTQTQMLRAFTANDGVMLEPKFISALY
DPNDQSVRKSQKEIVGNPVSKEAASVTRDHMVMVGTDPTYGTMHSTGKATVNVPGQNVALKSGAAEIADEKNGGYLTGSTN
NIFSVVSMHPAENPDFILYV
>JP_2x12
GTDGIITYEKDRLGNIVPGTEQVSQQTVDGKDVYTTISSPLQSFMETQMDAFQEKVKGKTATLVSAKTGEILATTQRPTFDADT
KEGITEDFVWRDILYQSNYEPGSTMKVMMLAAANNTFPGGEVFNSSELKIADATIRDWDVNEGLTGGRMMTFSQGFAHSSNV
GMTLLEQKMATWLDYLNRFKFGVPTRFGLTDEYAGQLPADNIVNIAMSAFGQGISVTQTQMLRAFTANDGVMLEPKFISALY
DPNDQSVRKSQKEIVGNPVSKEAASVTRDHMVMVGTDPTYGSMHSTGKATVNVPGQNVALKSGTAEIADEKNGGYLTGSTN
NIFSVVSMHPAENPDFILYV
>JP_2x13
GTDGIITYEKDRLGNIVPGTEQVSQQTVDGKDVYTTISSPLQSFMETQMDAFQEKVKGKTATLVSAKTGEILATTQRPTFDADT
KEGITEDFVWRDILYQSNYEPGSTMKVMMLAAANNTFPGGEVFNSSELKIADATIRDWDVNEGLTGGRMMTFSQGFAHSSNV
GMTFLEQKMATWLDYLNRFKFGVPTRFGLTDEYAGQLPADNIVNIAMSAFGQGISVTQTQMLRAFTANDGVMLEPKFISALY
DPNDQSVRKSQKEIVGNPVSKEAASVTRDHMVMVGTDPTYGTMHSTGKATVNVPGQNVALKSGTAEIADEKNGGYLTGSTN
NIFSVVSMHPAENPDFILYV
>JP_2x14
GTDGIITYEKDRLGNIVPGTEQVSQQTVDGKDVYTTISSTLQSFMETQMNAFQEKVKGKTATLVSAKTGEILATTQRPTFDADT
KEGLTKDFVWRDILYQSNYEPGSTMKVMTLAASNNTFPGGEYFNSSELKIADVTIRDWDVNDGLTTGRMMTFLQGFALSSNV
GMSLLEQKMTTWLDYLNRFKFGVPTRFGLTDEYAGQLPADNIVNIAQSSFGQGISVTQTQMIRAFTANDGVMLEPKFISAIYDT
NNQSVRKSQKEIVGKPVSEDAASLTRTNMILVGTDPLYGTMHQTGKPIITVPGQNVAVKSGTAQIADEKNGGYLVGSTNYIFS
VVTMNPAENPDFILYV
>JP_2x15
GTDGIITYEKDRLGNIVPGTEQVSQRTMDGKDVYTTISSPLQSFMETQMDAFQEKVKGKTATLVSAKTGEILATTQRPTFDADT
KEGLTKDFVWRDILYQSNYEPGSTMKVMTLASSNNTFPSGEYFNSSELKIADVTIRDWDVNDGLTTGRMMTFLQGFALSSNV
GMSLLEQKMTTWLDYLNRFKFGVPTRFGLTDEYAGQLPADNIVNIAQSSFGQGISVTQTQMLRAFTANDGVMLEPKFISAIYD
TNNQSVRKSQKEIVGKPVSEDAASLTRTNMILVGTDPIYGTMHYTGKPIITVPGQNVAVKSGTAQIADEKNGGYLVGSTNYIFS

VVTMNPAENPDFILYV
>JP_2x16
GTDGIITYEKDRVGNIVPGTELVSQQTVDGKDVYTTLSSPLQSFMETQMDAFLEKVKGKTATLVSAKTGEILATTQRPTFNADT
KEGITEDFVWRDILYQSNYEPGSAMKVITLASSNNTFPSGEYFNSSEFKIADATTRDWDVNEGLTTGGMMTFLQGFAHSSNVG
MSLLEQKMATWLDYLKRFKFGVPTRFGLTDEYAGQLPADNIVSIAQSSFGQGISVTQTQMLRAFTANDGVMLEPKFISAIYDT
NNQSVRKSQKEIVGKPVSEDTASLTRTNMILVGTDPLYGTMHYTGKPIITVPGQNVAVKSGTAQIADEKNGGYLVGSTNYIFSA
VTMNPAENPDFILYV
>JP_2x17
GTDGIITYEKDRLGNIVPGTELVSQQTVDGKDVYTTLSSPLQSFMETQMDAFLEKVKGKTATLVSAKTGEILATTQRPTFNADT
KEGITKDFVWRDILYQSNYEPGSAMKVMTLAAANNTFPSGEYFNSSEFKIADATTRDWDVNAGLTTGGMMTFLQGFAHSSNV
GMSLLEQKMATWLDYLKRFKFGVPTRFGLTDEYAGQLPADNIVSIAQSSFGQGISVTQTQMLRAFTANDGVMLEPKFISAIYD
TNNQSVRKSQKEIVGNPVPKEAASTTRNHMILVGTDPLYGTMHYTGKPIITVPGQNVAVKSGTAQIADEKNGGYLVGSTNYIF
SAVTMNPAENPDFILYV
>JP_2x18
GTDGIITYEKDRLGNIVPGTEQVSQQTVDGKDVYTTLSSPLQSFMETQMDAFLEKVKGKTATLVSAKTGEILATTQRPTFNADT
KEGITEDFVWRDILYQSNYEPGSAFKVMMLASSNNTFPSGEYFNSSEFKIADATTRDWDVNAGLTTGGMMTFLQGFAHSSNV
GTSLLEQKMATWLDYLKRFKFGVPTRFGLTDEYAGQLPADNIVSIAQSSFGQGISVTQTQMLRAFTANDGVMLEPKFISAIYDT
NNQSVRKSQKEIVGKPVSEDAASTTRNHMILVGTDPLYGTMHYTGKPIITVPGQNVAVKSGTAQIADEKNGGYLVGSTNYIFS
VVTMNPAENPDFILYV
>JP_2x19
GTDGIITYEKDRLGNIVPGTEQVSQQTVDGKDVYTTLSSPLQSFMETQMDAFLEKVKGKTATLVSAKTGEILATTQRPTFNADT
KEGITEDFVWRDILYQSNYEPGSAFKVMMLASSNNTFPSGEYFNSSEFKMADVTTRDWDVNGGLTTGGMMTFLQGFAHSSN
VGMSLLEQKMATWLDYLKRFKFGVPTRFGLTDEYAGQLPADNIVNIAQSSFGQGISVTQTQMLRAFTANDGVMLEPKFISAIY
DTNNQSVRKSQKEIVGKPVSEDAASTTRNHMILVGTDPLYGTMHYTGKPIITVPGQNVAVKSGTAQIADEKNGGYLVGSTNYI
FSVVTMNPAENPDFILYV
>JP_2x20
GTDGIITYEKDRLGNIVPGTEQISQQTVDGKDVYTTISSPLQSFMETQMDAFQEKVKGKTATLVSAKTGEILATTQRPTFNADT
KEGITKDFVWRDILYQSNYEPGSAFKVMMLASSNNTFPSGEYFNSSEFKIADATTRDWDVNAGLTTGGMMTFLQGFAHSSNV
GTSLLEQKMATWLDYLKRFKFGVPTRFGLTDEYAGQLPADNIVSIAQSSFGQGISVTQTQMLRAFTANDGVMLEPKFISAIYDT
NNQSVRKSQKEIVGNPVSKEAASTTRNHMILVGTDPLYGTMHYTGKPIITVPGQNVAVKSGTAQIADEKNGGYLVGSTNYIFS

VVTMNPAENPDFILYV
>JP_2x21
GTDGIITYEKDRLGNIVPGTELVSQQTVDGKDVYTTLSSPLQSFMETQMDAFLEKEKGKTATLVSAKTGEILATTQRPTFNADT
KEGITEDFVWRDILYQSNYEPGSGMKAMTLASSNNTFPSGEYFNSSEFKIADVTTRDWDVNEGLTTGGMMTFLQGFAHSSNV
GMSLLEQKMATWLDYLKRFKFGVPTRFGLTDEYAGQLPADNIVSIAQSSFGQGISVTQTQMLRAFTANDGVMLEPKFISAIYD
TNNQSVRKSQKEIVGNPVSKEAASTTRNHMILVGTDPLYGTMHYTGKPIITVPGQNVAVKSGTAQIADEKNGGYLVGSTNYIF
SVVTMNPAENPDFILYV
>JP_2x22
GKDGIITYEKDRLGNIVPGTEQVSQQTVDGKDVYTTLSSPLQSFMETQMDAFLEKVKGKTATLVSAKTGEILATTQRPTFNAD
TKEGITEDFVWRDILYQSNYEPGSAMKVMTLASSNNTFPSGEYFNSSEFKIADATTRDWDVNDGLTTGGMMTFLQGFAHSSN
VGMSLLEQKMATWLDYLKRFKFGVPTRFGLTDEYAGQLPADNIVSIAQSSFGQGISVTQTQMLRAFTANDGVMLEPKFISAIY
DTNNQSVRKSQKEIVGNPVSKEAASTTRNHMILVGTDPLYGTMHYTGKTIITVPGQNVAVKSGTAQIADEKNGGYLVGSTNYI
FSVVTMNPAENPDFILYV
>JP_2x23
GTDGIITYEKDRLGNIVPGTEQVSQRTMDGKDVYTTISSPLQSFMETQMDAFLEKVKGKTATLVSAKTGEILATTQRPTFNADT
KEGITKDFVWRDILYQSNYEPGSTMKVMTLAAANNTFPGGEVFDSSELKIADVTTRDWDVNEGLTGGRMMTFSQGFALSSNV
GMTLLEQKMATWLDYLNRFKFGVPTRFGLTDEYAGQLPADNIVNIAQSSFGQGISVTQTQMIRAFTANDGVMLEPKFISAIYD
PNDQTARKSQKEIVGNPVSKDAASLTRTHMVLVGTDPVYGTMHSTGKPTVTVPGQNIALKSGTAQIADEKNGGYLVGTTNHI
FSAVSMNPAENPDFILYV
>JP_2x24
GTDGIITYEKDRLGNIVPGTEQVSQRTMDGKDVYTTISSPLQSFMETQMDAFQEKVKGKTATLVSAKTGEILATTQRPTFDADT
KEGITKDFVWRDILYQSNYEPGSTMKVMTLAAANNTFPGGEVFNSSELKIADVTIRDWDVNDGLTTGRMMTFLQGFALSSNV
GMSLLEQKMATWLDYLNRFKFGVPTRFGLTDEYAGQLPADNIVNIAQSSFGQGISVTQTQMLRAFTANDGVMLEPKFISAIYD
PNDQTARKSQKEIVGNPVSKDAASLTRTNMVLVGTDPVYGTMHQTGKPIITVPGQNVAVKSGTAQIADEKNGGYLVGSTNYI
FSVVTMNPAENPDFILYV
>JP_2x25
GTDGIITYEKDRLGNIVPGTEQVSQQTVDGKDVYTTISSPLQSFMETQMDAFQEKVKGKTATLVSAKTGEILATTQRPTFDADT
KEGLTKDFVWRDILYQSNYEPGSAMKVMTLASSNNTFPSGEYFNSSEFKIADATTRDWDVNDGLTTGGMMTFLQGFAHSSNV
GMSLLEQKMATWLDYLKRFKFGVPTRFGLTDEYAGQLPADNIVSIAQSSFGQGISVTQTQMLRAFTANDGVMLEPKFISAIYD
TNNQSVRKSQKEIVGNPVSKEAASTTRNHMILVGTDPLYGTMHYTGKPIITVPGQNVAVKSGTAQIADEKNGGYLVGSTNYIF

SAVTMNPAENPDFILYV
>JP_2x26
GKDGIITYEKDRLGNIVPGTEQVSQQTVDGKDVYTTISSTLQSFMETQMDAFLEKLKGKTATLVSAKTGEILATTQRPTFNADT
KEGITEDFVWRDILYQSNYEPGSAMKVMTLASSNNTFPSGEYFNSSEFKIADATTRDWDVNDGLTTGGMMTFLQGFAHSSNV
GMSLLEQKMATWLDYLSRFKFGVPTRFGLTDEYAGQLPADNIVSIAQSSFGQGISVTQTQMLRAFTANDGVMLEPKFISAIYDT
NNQSVRKSQKEIVGNPVSKEAASTTRNHMILVGTDPLYGTMHYTGKPIITVPGQNVAVKSGTAQIADEKNGGYLVGSTNYIFS
VVTMNPAENPDFILYV
>JP_2x27
GTDGIITYEKDRLGNIVPGTEQVSQRTMDGKDVYTTISSPLQSFMETQMDAFQEKVKGKTATLVSAKTGEILATTQRPTFDADT
KEGITEDFVWRDILYQSNYEPGSTMKVMMLAAANNTFPGGEVFNSSELKIADATIRDWDVNEGLTGGRMMTFSQGFAHSSNV
GMTLLEQKMATWLDYLNRFKFGVPTRFGLTDEYAGQLPADNIVNIAQSSFGQGISVTQTQMLRAFTANDGVMLEPKFISALY
DPNDQSVRKSQKEIVGNPVSKAAASSTREHMVMVGTDPVYGTMHSTGKPNVNVPGQNVALKSGTAEIADEKNGGYLTGETN
NIFSVVSMHPAENPDFILYV
>JP_2x28
GTDGIITYEKDRLGNIVPGTEQASQHTVDGKDVYTTLSSPLQSFMETQMDAFQEKLKGKTATLVSAKTGEILATTQRPTFNADT
KDGITKDFVWRDILYQSNYEPGSAMKVMTLASANNTFPGGEYFNSSELKIADATIRDWDVNDGLTTGGMMTFSQGFAHSSNV
GMSLLEQKMATWLDYLNRFKFGVPTRFGLTDEYTGQLPADNIVNIAMSAFGQGISVTQTQMLRAFTANDGVMLEPKFISALY
DPNDQSVRKSQKEIVGNPVSKAAASSTREHMVMVGTDPVYGTMHSTGKPNVNVPGQNVALKSGTAQIADEKNGGYLTGETN
YIFSVVSMHPAENPDFILYV
>JP_2x29
GTDGIITYEKDRLGNIVPGTEQASQHTVDGKDVYTTLSSPLQSFMETQMDAFQEKEKGKTATLVSAKTGEILATTQRPTFNADT
KDGITKDFVWRDILYQSNYEPGSAMKVMTLASANNTFPGGEYFNSSELKIADATIRDWDVNDGLTTGGMMTFSQGFAHSSNV
GMSLLEQKMATWLDYLNRFKFGVPTRFGLTDEYTGQLPADNIVNIAMSAFGQGISVTQTQMLRAFTANDGVMLEPKFISALY
DPNDQSVRKSQKEIVGNPVSKAAASSTREHMVMVGTDPVYGTMHSTGKPNVNVPGQNVALKSGTAQIADEKNGGYLTGETN
YIFSVVSMHPAENPDFILYV
>JP_2x30
GTDGIITYEKNRLGNIVPGTEQASQHTVDGKDVYTTLSSPLQSFMETQMDAFQEKVKGKTATLVSAKTGEILATTQRPTFNAD
TKDGITKDFVWRDILYQSNYEPGSAMKVMTLASANNTFPGGEYFNSSELKIADATIRDWDVNDGLTTGGMMTFSQGFAHSSN
VGMSLLEQKMATWLDYLNRFKFGVPTRFGLTDEYTGQLPADNIVNIAMSAFGQGISVTQTQMLRAFTANDGVMLEPKFISAL
YDPNDQSVRKSQKEIVGNPVSKAAASSTREHMVMVGTDPVYGTMHSTGKPNVNVPGQNVALKSGTAQIADEKNGGYLTGET

NYIFSVVSMHPAENPDFILYV
>JP_2x31
GTDGIITYEKDRLGNIVPGTEQASQHTVDGKDVYTTLSSPLQSFMETQMDAFQEKVKGKTATLVSAKTGEILATTQRPTFNAD
TKDGITKDFVWRDILYQSNYEPGSAMKVMTLASANNTFPGGEYFNSSELKIADATIRDWDVNDGLTTGGMMTFSQGFAHSSN
VGMSLLEQKMATWLDYLNRFKFGVPTRFGLTDEYTGQLPADNIVNIAMSAFGQGISVTQTQMLRAFTANDGVMLEPKFISAL
YDPNDQSVRKSQKEIVGNPVSKAAASSTREHMVMVGTDPVYGTMHSTGKPNVNVPGQNVALKSGTAQIADEKNGGYLTGET
NYIFSVVSMHPAENPDFILYV
>JP_2x32
GTDGIITYEKDRLGNIVPGTEQASQHTVDGKDVYTTLSSPLQSFMETQMDAFQEKVKGKTATLVSAKTGEILATTQRPTFNAD
TKDGITKDFVWRDILYQSNYEPGSAMKVMTLASANNTFPGGEYFNSSELKIADATIRDWDVNDGLTTGGMMTFSQGFAHSSN
VGMSLLEQKMATWLDYLNRFKFGVPTRFGLTDEYTGQLPADNIVNIAMSAFGQGISVTHTQMLRAFTANDGVMLEPKFISAL
YDPNDQSVRKSQKEIVGNPVSKAAASSTREHMVMVGTDPVYGTMHSTGKPNVNVPGQNVALKSGTAQIADEKNGGYLTGET
NYIFSVVSMHPAENPDFILYV
>JP_2x33
GTDGIITYEKDRLGNIVPGTEQVSQQTVDGKDVYTTISSPLQSFMETQMDAFQEKVKGKTATLVSAKTGEILATTQRPTFDADT
KEGLTKDFVWRDILYQSNYEPGSTMKVMTLAAANNTFPGGEVFDSSELKIADVTIRDWDVNEGLTGGRMMTLSQGFALSSNV
GMTLLEQKMATWLDYLNRFKFGVPTRFGLTDEYAGQLPADNIVNIAQSSFGQGISVTQTQMLRAFTANDGVMLEPKFISALY
DPNDQSVRKSQKEIVGNPVSKDAASLTRTNMVLVGTDPVYGTMHSTGKPTVTVPGQNVALKSGTAQIADEKNGGYLVGLTD
YIFSAVSMSPAENPDFILYV
>JP_2x34
GKDGIITYEKDRLGNIVPGTEQVFQQTVDGKDVYTTISSTLQSFMETQMNAFQEKVKGKTATLVSAKTGEILATTQRPTFDADT
KEGLTKDFVWRDILYQSNYEPGSTMKVMTLAAANNTFPGGEVFDSSELKIADVTIRDWDVNEGLTGGRMMTFSQGFVLSSNV
GMTLLEQKMATWLDYLNRFKFGVPTRFGLTDEYAGQLPADNIVNIAQSSFGQGISVTQTQMIRAFTANDGVMLEPKFISAIYD
PNDQTARKSQKEIVGNPVSKDAASLTRTNMVLVGTDPVYGTMHSTGKPTVTVPGQNVALKSGTAQIADEKNGGYLVGLTDYI
FSAVSMSPAENPDFILYV
>JP_2x35
GTDGIITYEKDRLGNIVPGTEQVSQQTVNGKDVYTTLSSPLQSFMETQMNAFQEKVKGKTATLVSAKTGEILATTQRPTFDAD
TKEGLTKDFVWRDILYQSNYEPGSTMKVMTLAAANNTFPGGEVFDSSELKIADVTIRDWDVNEGLTGGRMMTFSQGFALSSN
VGMTLLEQKMATWLDYLNRFKFGVPTRFGLTDEYAGQLPADNIVNIAQSSFGQGISVTQTQMIRAFTANDGVMLEPKFISAIY
DPNDQTARKSQKEIVGNPVSKDAASLTRTHMVLVGTDPVYGTMHSTGKPTVTVPGQNVALKSGTAQIADEKNGGYLVGLTN

YIFSAVSMSPAENPDFILYV
>JP_2x36
GTDGIITYEKDRLGNIVPGTEQVSQQTVNGKDVYTTLSSPLQSFMETQMDAFLEKVKGKTATLVSAKTGEILATTQRPTFNADT
KEGITKDFVWRDILYQSNYEPGSTMKVMTLAAANNTFPGGEVFDSSELKIADVTTRDWDVNEGLTGGRMMTFSQGFALSSNV
GMTLLEQKMATWLDYLNRFKFGVPTRFGLTDEYAGQLPADNIVNIAQSSFGQGISVTQTQMIRAFTANDGVMLEPKFISAIYD
PNDQTARKSQKEIVGNPVSKDAASLTRTHMVLVGTDPVYGTMHSTGKPTVTVPGQNIALKSGTAQIADEKNGGYLVGTTNHI
FSAVSMNPAENPDFILYV
>JP_2x37
GKDGIITYEKDRLGNIVPGTEQVSQQTVDGKDVYTTISSTLQSFMETQMNAFQEKVKGKTATLVSAKTGEILATTQRPTFDADT
KEGLTKDFVWRDILYQSNYEPGSTMKVMTLAAANNTFPGGEVFDSSELKIADVTIRDWDVNEGLTGGRMMTFSQGFVLSSNV
GMTLLEQKMATWLDYLNRFKFGVPTRFGLTDEYAGQLPADNIVNIAQSSFGQGISVTQTQMIRAFTANDGVMLEPKFITALYD
PNNQSVRKSQKEIVGNPVSKDAASLTRTHMLLVGTDPVYGTMHSTGKPTVTVPGQNVALKSGTAQIADEKNGGYLIGSTNYIF
SAVSMNPAENPDFILYV
>JP_2x38
GTDGIITYEKDRLGNIVPGTEQVSQQTVDGKDVYTTISSTLQSFMETQMDAFQEKVKGKTATLVSAKTGEILATTQRPTFDADT
KEGITEDFVWRDILYQSNYEPGSTMKVMTLAAANNTFPGGEVFNSSELKIADATIRDWDVNEGLTGGRMMTFSQGFAHSSNV
GMTLLEQKMATWLDYLNRFKFGVPTRFGLTDEYAGQLPADNIVNIAQSSFGQGISVTQTQMIRAFTANDGVMLEPKFISAIYD
PNDQTARKSQKEIVGNPVSKDAASLTRTNMVLVGTDAVYGTMHSTGKPTVTVPGQNVALKSGTAEIADEKNGGYLVGSTNNI
FSVVAMNPAENPDFILYV
>JP_2x39
GTDGIITYEKDRVGNIVPGTELVSQQTVDGKDVYTTLSSPLQSFMETQMDAFLEKVKGKTATLVSAKTGEILATTQRPTFNADT
KEGITEDFVWRDILYQSNYEPGSAMKVMTLASSNNTFPSGEYFNSSEFKIADATTRDWDVNAGLTTGGMMTFLQGFAHSSNV
GMSLLEQKMATWLDYLKRFKFGVPTRFGLTDEYAGQLPADNIVSIAQSSFGQGISVTQTQMLRAFTANDGVMLEPKFISAIYD
TNNQSVRKSQKEIVGNPVSKEAASTTRNHMILVGTDPLYGTMHYTGKPIITVPGQNVAVKSGTAQIADEKNGGYLVGSTNYIF
SAVTMNPAENPDFILYV
>JP_2x40
GTDGIITYEKDRVGNIVPGTELVSQQTVDGKDVYTTLSSPLQSFMETQMDAFLEKVKGKTATLVSAKTGEILATTQRPTFNADT
KEGITEDFVWRDILYQSNYEPGSAMKVMTLASSNNTFPSGEYFNSSEFKIADATTRDWDVNEGLTTGGMMTFLQGFAHSSNV
GMSLLEQKMATWLDYLKRFKFGVPTRFGLTDEYAGQLPADNIVSIAQSSFGQGISVTQTQMLRAFTANDGVMLEPKFISAIYD
TNNQSVRKSQKEIVGNPVSKEVASTTRNHMILVGTDPIYGTMHYTGKPIITVPGQNVAVKSGTAQIADEKNGGYLVGSTNYIFS

VVTMNPAENPDFILYV
>JP_2x41
GKDGIITYEKDRLGNIVPGTEQVSQQTVDGKDVYTTLSSPLQSFMETQMDAFLEKVKGKTATLVSAKTGEILATTQRPTFNAD
TKEGITEDFVWRDILYQSNYEPGSAMKVMTLASSNNTFPSGEYFNSSEFKIADATTRDWDVNEGLTTGGMMTFLQGFAHSSN
VGMSLLEQKMATWLDYLKRFKFGVPTRFGLTDEYAGQLPADNIVSIAQSSFGQGISVTQTQMLRAFTANDGVMLEPKFISAIY
DTNNQSVRKSQKEIVGNPVSKEAASTTRNHMILVGTDPLYGTMHYTGKPIITVPGQNVAVKSGTAQIADEKNGGYLVGSTNYI
FSAVTMNPAENPDFILYV
>JP_2x42
GTDGIITYEKDRLGNIVPGTEQVSQQTVDGKDVYTTLSSPLQSFMETQMDAFLEKVKGKTATLVSAKTGEILATTQRPTFNADT
KEGITEDFVWRDILYQSNYEPGSAMKVMTLASSNNTFPSGEYFNSSEFKIADATTRDWDVNDGLTTGGMMTFLQGFAHSSNV
GMSLLEQKMATWLDYLKRFKFGVPTRFGLTDEYAGQLPADNIVSIAQSSFGQGISVTQTQMLRAFTANDGVMLEPKFISAIYD
TNNQSVRKSQKEIVGNPVSKEAASTTRNHMILVGTDPLYGTMHYTGKPIITVPGQNVAVKSGTAQIADEKNGGYLVGSTNYIF
SVVTMNPAENPDFILYV
>JP_2x43
GKDGIITYEKDRLGNIVPGTEQVSQQTVDGKDVYTTLSSPLQSFMETQMDAFLEKVKGKTATLVSAKTGEILATTQRPTFNAD
TKEGITEDFVWRDILYQSNYEPGSAMKVMTLASSNNTFPSGEYFNSSEFKIADATTRDWDVNDGLTTGGMMTFLQGFAHSSN
VGMSLLEQKMATWLDYLNRFKFGVPTRFGLTDEYAGQLPADNIVSIAQSSFGQGISVTQTQMLRAFTANDGVMLEPKFISAIY
DTNNQSVRKSQKEIVGNPVSKEAASTTRNHMILVGTDPLYGTMHYTGKPIITVPGQNVAVKSGTAQIADEKNGGYLVGSTNYI
FSVVTMNPAENPDFILYV
>JP_2x44
GKDGIITYEKDRVGNIVPGTELVSQQTVDGKDVYTTLSSPLQSFMETQMDAFLEKVKGKTATLVSAKTGEILATTQRPTFNAD
TKEGITEDFVWRDILYQSNYEPGSAFKVMMLASSNNTFPSGEYFNSSEFKMADVTTRDWDVNGGLTTGGMMTFLQGFAHSSN
VGTSLLEQKMATWLDYLKRFKFGVPTRFGLTDEYAGQLPADNIVSIAQSSFGQGISVTQTQMLRAFTANDGVMLEPKFISAIYD
TNNQSVRKSQKEIVGNPVSKEAASTTRNHMILVGTDPLYGTMHYTGKPIITVPGQNVAVKSGTAQIADEKNGGYLVGSTNYIF
SVVTMNPAENPDFILYV
>JP_2x45
GKDGIITYEKDRLGNIVPGTEQVSQQTVDGKDVYTTLSSPLQSFMETQMDAFLEKVKGKTATLVSAKTGEILATTQRPTFNAD
TKEGITEDFVWRDILYQSNYEPGSAFKVMMLASSNNTFPSGEYFNSSEFKMADVTTRDWDVNGGLTTGGMMTFLQGFAHSSN
VGTSLLEQKMATWLDYLKRFKFGVPTRFGLTDEYAGQLPADNIVSIAQSSFGQGISVTQTQMLRAFTANDGVMLEPKFISAIYD
TNNQSVRKSQKEIVGNPVSKEAASTTRNHMILVGTDPLYGTMHYTGKPIITVPGQNVAVKSGTAQIADEKNGGYLVGSTNYIF

SVVTMNPAENPDFILYV
>JP_2x46
GKDGIITYEKDRLGNIVPGTEQVSQQTVDGKDVYTTLSSPLQSFMETQMDAFLEKVKGKTATLVSAKTGEILATTQRPTFNAD
TKEGITEDFVWRDILYQSNYEPGSAFKVMMLASSNNTFPSGEYFNSSEFKLADATTRDWDVNAGLTTGGMMTFLQGFVHSSN
VATSLLEQKMATWLDYLKRFKFGVPTRFGLTDEYAGQLPADNIVSIAQSSFGQGISVTQTQMLRAFTANDGVMLEPKFISAIYD
TNNQSVRKSQKEIVGNPVSKEAASTTRNHMILVGTDPLYGTMHYTGKPIITVPGQNVAVKSGTAQIADEKNGGYLVGSTNYIF
SVVTMNPAENPDFILYV
>JP_2x47
GKDGIITYEKDRLGNIVPGTEQVSQQTVDGKDVYTTLSSPLQSFMETQMDAFLEKLKGKTATLVSAKTGEILATTQRPTFNADT
KEGITEDFVWRDILYQSNYEPGSAFKVMMLASSNNTFPSGEYFNSSEFKIADATTRDWDVNEGLTTGGMMTFLQGFAHSSNV
GTSLLEQKMATWLDYLKRFKFGVPTRFGLTDEYAGQLPADNIVSIAQSSFGQGISVTQTQMLRAFTANDGVMLEPKFISAIYDT
NNQSVRKSQKEIVGNPVSKEAASTTRNHMILVGTDPLYGTMHYTGKPIITVPGQNVAVKSGTAQIADEKNGGYLVGSTNYIFS
VVTMNPAENPDFILYV
>JP_2x48
GKDGIITYEKDRLGNIVPGTEQVSQQTVDGKDVYTTLSSPLQSFMETQMDAFLEKVKGKTATLVSAKTGEILATTQRPTFNAD
TKEGITEDFVWRDILYQSNYEPGSAFKVMMLASSNNTFPSGEYFNSSEFKLADATTRDWDVNGGLTTGGMMTFLQGFAHSSN
VGMSLLEQKMATWLDYLKRFKFGVPTRFGLTDEYAGQLPADNIVSIAQSSFGQGISVTQTQMLRAFTANDGVMLEPKFISAIY
DTNNQSVRKSQKEIVGKPVSEDAASTTRNHMILVGTDPLYGTMHYTGKPIITVPGQNVAVKSGTAQIADEKNGGYLVGSTNYI
FSVVTMNPAENPDFILYV
>JP_2x49
GTDGIITYEKDRLGNIVPGTELVSQQTVDGKDVYTTLSSPLQSFMETQMDAFLEKVKGKTATLVSAKTGEILATTQRPTFNADT
KEGITEDFVWRDILYQSNYEPGSAMKVMTLASSNNTFPSGEYFNSSEFKIADATTRDWDVNEGLTTGGMMTFLQGFAHSSNV
GMSLLEQKMATWLDYLNRFKFGVPTRFGLTDEYAGQLPADNIVSIAQSSFGQGISVTQTQMLRAFTANDGVMLEPKFISAIYD
TNNQSVRKSQKEIVGNPVSKEAASTTRNHMILVGTDPLYGTMHYTGKPIITVPGQNVAVKSGTAQIADEKNGGYLVGSTNYIF
SVVTMNPAENPDFILYV
>JP_2x50
GTDGIITYEKDRLGNIVPGTEQVSQQTVDGKDVYTTISSTLQSFMETQMDAFLEKVKGKTATLVSAKTGEILATTQRPTFNADT
KEGITEDFVWRDILYQSNYEPGSAMKVMTLASSNNTFPSGEYFNSSEFKIADATTRDWDVNDGLTTGGMMTFLQGFAHSSNV
GMSLLEQKMATWLDYLSRFKFGVPTRFGLTDEYAGQLPADNIVSIAQSSFGQGISVTQTQMLRAFTANDGVMLEPKFISAIYDT
NNQSVRKSQKEIVGNPVSKEAASTTRNHMILVGTDPLYGTMHYTGKPIITVPGQNVAVKSGTAQIADEKNGGYLVGSTNYIFS

VVTMNPAENPDFILYV
>JP_2x51
GTDGIITYEKDRLGNIVPGTEQVSQQTVDGKDVYTTISSTLQSFMETQMDAFQGKLKGKTATLVSAKTGEILATTQRPTFNADT
KEGITEDFVWRDILYQSNYEPGSAMKVMTLASSNNTFPSGEYFNSSEFKIADATTRDWDVNEGLTTGGMMTFLQGFAHSSNV
GMSLLEQKMATWLDYLKRFKFGVPTRFGLTDEYAGQLPADNIVSIAQSSFGQGISVTQTQMLRAFTANDGVMLEPKFISAIYD
TNNQSVRKSQKEIVGNPVSKEAASTTRNHMILVGTDPLYGTMHYTGKPIITVPGQNVAVKSGTAQIADEKNGGYLVGSTNYIF
SVVTMNPAENPDFILYV
>JP_2x52
GTDGIITYEKDRLGNIVPGTEQVSQQTVDGKDVYTTISSPLQSFMETQMDAFQEKLKGKTATLVSAKTGEILATTQRPTFNADT
KEGITEDFVWRDILYQSNYEPGSAFKVMMLASSNNTFPSGEYFNSSEFKIADATTRDWDVNEGLTTGGMMTFLQGFAHSSNV
GTSLLEQKMATWLDYLKRFKFGVPTRFGLTDEYAGQLPADNIVSIAQSSFGQGISVTQTQMLRAFTANDGVMLEPKFISAIYDT
NNQSVRKSQKEIVGNPVSKEAASTTRNHMILVGTDPLYGTMHYTGKPIITVPGQNVAVKSGTAQIADEKNGGYLVGSTNYIFS
VVTMNPAENPDFILYV
>JP_2x53
GTDGIITYEKDRLGNIVPGTEQVSQRTMDGKDVYTTLSSPLQSFMETQMDAFLEKVKGKTATLVSAKTGEILATTQRPTFNAD
TKEGITEDFVWRDILYQSNYEPGSAMKVMTLASSNNTFPSGEYFNSSEFKIADATTRDWDVNEGLTTGGMMTFLQGFAHSSN
VGMSLLEQKMATWLDYLKRFKFGVPTRFGLTDEYAGQLPADNIVSIAQSSFGQGISVTQTQMLRAFTANDGVMLEPKFISAIY
DTNNQSVRKSQKEIVGNPVSKEAASTTRNHMILVGTDPLYGTMHYTGKPIITVPGQNVAVKSGTAQIADEKNGGYLVGSTNYI
FSAVTMNPAENPDFILYV
>JP_2x54
GTDGIITYEKDRLGNIVPGTEQVSQRTMDGKDVYTTLSSPLQSFMETQMDAFLEKVKGKTATLVSAKTGEILATTQRPTFNAD
TKEGITEDFVWRDILYQSNYEPGSAMKVMTLASSNNTFPSGEYFNSSEFKIADATTRDWDVNDGLTTGGMMTFLQGFAHSSN
VGMSLLEQKMATWLDYLKRFKFGVPTRFGLTDEYAGQLPADNIVSIAQSSFGQGISVTQTQMLRAFTANDGVMLEPKFISAIY
DTNNQSVRKSQKEIVGNPVSKEAASTTRNHMILVGTDPLYGTMHYTGKPIITVPGQNVAVKSGTAQIADEKNGGYLVGSTNYI
FSAVTMNPAENPDFILYV
>JP_2x55
GTDGIITYEKDRLGNIVPGTEQVSQRTMDGKDVYTTISSPLQSFMETQMDAFQEKLKGKTATLVSAKTGEILATTQRPTFNADT
KEGITEDFVWRDILYQSNYEPGSAMKVMTLASSNNTFPSGEYFNSSEFKIADATTRDWDVNAGLTTGGMMTFLQGFAHSSNV
GMSLLEQKMATWLDYLKRFKFGVPTRFGLTDEYAGQLPADNIVSIAQSSFGQGISVTQTQMLRAFTANDGVMLEPKFISAIYD
TNNQSVRKSQKEIVGNPVSKEAASTTRNHMILVGTDPLYGTMHYTGKPIITVPGQNVAVKSGTAQIADEKNGGYLVGSTNYIF

SAVTMNPAENPDFILYV
>JP_2x56
GTDGIITYEKDRLGNIVPGTEQVSQRTMDGKDVYTTISSPLQSFMETQMDAFQEKVKGKTATLVSAKTGEILATTQRPTFDADT
KEGITEDFVWRDILYQSNYEPGSTMKVMMLAAANNTFPGGEVFNSSELKIADATIRDWDVNEGLTVGGMMTFLQGFAHSSNV
GMSLLEQKMATWLDYLNRFKFGVPTRFGLTDEYAGQLPADNIVSIAQSSFGQGISVTQTQMLRAFTANDGVMLEPKFISAIYD
TNNQSVRKSQKEIVGKPVSEDAASLTRTNMILVGTDPLYGTMHYTGKPIITVPGQNVAVKSGTAQIADEKNGGYLVGSTNYIFS
VVTMNPAENPDFILYV
>JP_2x57
GKDGIITYEKDRLGNIVPGTEQVSQQTVDGKDVYTTLSSPLQSFMETQMDAFLEKVKGKTATLVSAKTGEILATTQRPTFNAD
TKEGITEDFVWRDILYQSNYEPGSTMKVMTLAAANNTFPGGEVFNSSELKIADVTIRDWDVNDGLTTGRMMTFLQGFALSSN
VGMSLLEQKMTTWLDYLNRFKFGVPTRFGLTDEYSGQLPEDNIVNIAQSSFGQGISVTQTQMLRAFTANDGVMLEPKFISAIY
DTNNQSVRKSQKEIVGKPVSEDAASLTRTNMILVGTDPLYGTMHQTGKPIITVPGQNVAVKSGTAQIADEKNGGYLVGSTNYI
FSVVTMNPAENPDFILYV
>JP_2x58
GTDGIITYEKDRLGNIVPGTEQVSQRTMDGKDVYTTISSPLQSFMETQMDAFQEKVKGKTATLVSAKTGEILATTQRPTFDADT
KEGITEDFVWRDILYQSNYEPGSAMKVMMLAAANNTFPGGEVFNSSELKIADATIRDWDVNEGLTGGRMMTFSQGFAHSSNV
GMTLLEQKMATWLDYLNRFKFGVPTRFGLTDEYAGQLPADNIVNIAQSSFGQGISVTQTQMLRAFTANDGVMLEPKFISALY
DPNDQSVRKSQKEIVGNPVSKAAASSTREHMVMVGTDPVYGTMHSTGKPNVNVPGQNVALKSGTAEIADEKNGGYLTGETN
NIFSVVSMHPAENPDFILYV
>JP_2x59
GTDGIITYEKDRLGNIVPGTEQVSQRTMDGKDVYTTISSPLQSFMETQMDAFQEKVKGKYMTATLVSAKTGEILATTQRPTFD
ADTKEGITEDFVWRDILYQSNYEPGSAMKVMTLASSIDNNTFPSGEYFNSSEFKIADATTRDWDVNEGLTTGGMMTFLQGFAH
SSNVGMSLLEQKMGDATWLDYLKRFKFGVPTRFGLTDEYAGQLPADNIVNIAQSSFGQGISVTQTQMLRAFTAIANDGVMLE
PKFISAIYDTNNQSVRKSQKEIVGNPVSKEAASTTRNHMILVGTDPLYGTMYNHYTGKPIITVPGQNVAVKSGTAQIADEKNGG
YLVGSTNYIFSAVTMNPAENPDFILYV
>JP_2x60
GKDGIITYEKDRLGNIVPGTEQVSQQTVDGKDVYTTLSSPLQSFMETQMDAFLEKVKGKYMTATLVSAKTGEILATTQRPTFN
ADTKEGITEDFVWRDILYQSNYEPGSAFKVMMLASSIDNNTFPSGEYFNSSEFKLADATTRDWDVNAGLTTGGMMTFLQGFV
HSSNVGTSLLEQKMGDATWLDYLKRFKFGVPTRFGLTDEYAGQLPADNIVSIAQSSFGQGISVTQTQMLRAFTAIANDGVMLE
PKFISAIYDTNNQSVRKSQKEIVGNPVSKEAASTTRNHMILVGTDPLYGTMYNHYTGKPIITVPGQNVAVKSGTAQIADEKNGG

YLVGSTNYIFSVVTMNPAENPDFILYV
>JP_2x61
GTDGIITYEKDRVGNIVPGTELVSQQTVDGKDVYTTLSSPLQSFMETQMDAFLEKVKGKYMTATLVSAKTGEILATTQRPTFN
ADTKEGITEDFVWRVILYQSNYEPGSAMKVMTLASSIDNNTFPSGEYFNSSEFKIADATTRDWDVNEGLTTGGMMTFLQGFAH
SSNVGMSLLEQKMGDATWLDYLKRFKFGVPTRFGLTDEYAGQLPADNIVSIAQSSFGQGISVTQTQMLRAFTAIANDGVMLEP
KFISAIYDTNNQSVRKSQKEIVGKPVSEDTASLTRTNMILVGTDPLYGTMYNHYTGKPIITVPGQNVAVKSGTAQIADEKNGGY
LVGSTNYIFSAVTMNPAENPDFILYV
>JP_2x62
GTDGIITYEKDRLGNIVPGTEQVSQQTVDGKDVYTTLSSPLQSFMETQMDAFQEKLKGKYMTATLVSAKTGEILATTQRPTFN
ADTKEGITEDFVWRDILYQSNYEPGSAFKVMMLASSIDNNTFPSGEYFNSSEFKLADATTRDWDVNAGLTTGGMMTFLQGFV
HSSNVGTSLLEQKMGDATWLDYLKRFKFGVPTRFGLTDEYAGQLPADNIVSIAQSSFGQGISVTQTQMLRAFTAIANDGVMLE
PKFISAIYDTNNQSVRKSQKEIVGNPVSKEAASTTRNHMILVGTDPLYGTMYNHYTGKPIITVPGQNVAVKSGTAQIADEKNGG
YLVGSTNYIFSVVTMNPAENPDFILYV
>JP2x_63
GTDGIITYEKDRVGNIVPGTEQVSQQTVDGKDVYTTLSSPLQSFMETQMDAFLQKVKGKYMTATLVSAKTGEILATTQRPTFN
ADTKEGITEDFVWRDILYQSNYEPGSAFKVMMLASSIDNNTFPSGEYFNSSEFKMADVTTRDWDVNGGLTTGGMMTFLQGFA
HSSNVGTSLLEQKMGDATWLDYLKRFKFGVPTRFGLTDEYAGQLPADNIVSIAQSSFGQGISVTQTQMLRAFTAIANDGVMLE
PKFISAIYDTNNQSVRKSQKEIVGNPVSKEAASTTRNHMILVGTDPLYGTMYNHYTGKPIITVPGQNVAVKSGTAQIADEKNGG
YLVGSTNYIFSVVTMNPAENPDFILYV

Supplementary Figures

(a)

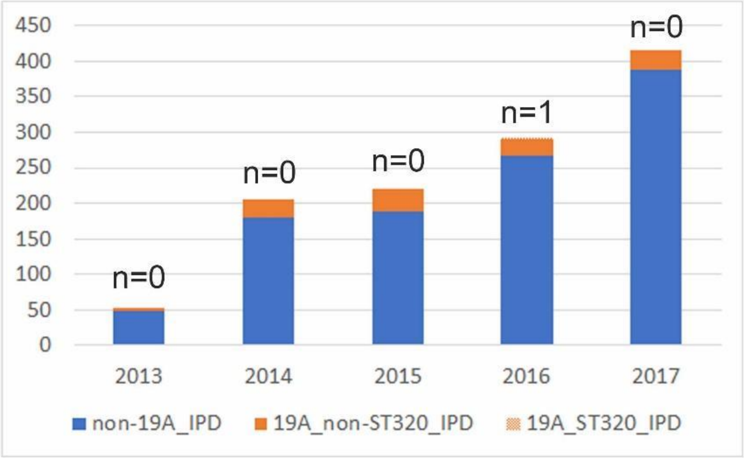

(b)

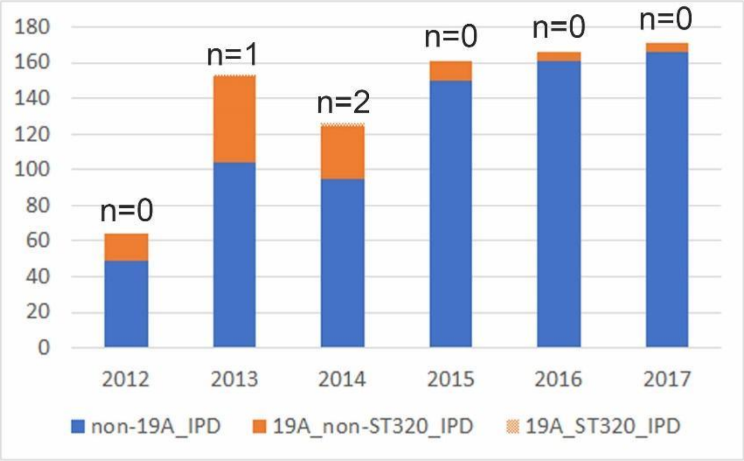

(c)

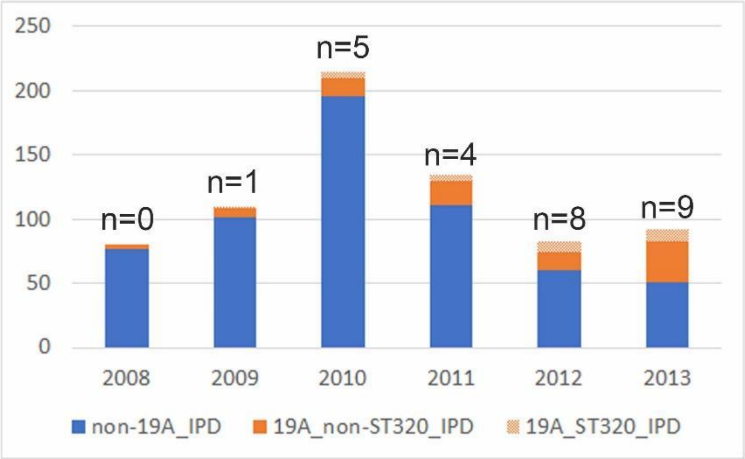

Figure S1. Serotype 19A and its genotype prevalence data in nationwide surveillance studies in Japan used in this study. Numbers shown on top of each bar indicate the numbers of 19A-ST320 IPD isolates in each year. (a) Adult invasive pneumococcal disease surveillance study conducted by Fukushima et al. and Shimbashi et al. (4) (b) Pediatric invasive pneumococcal disease surveillance study conducted by Nakano et al. (1, 2). (c) Pediatric invasive pneumococcal disease surveillance study conducted by Suga et al. (3).

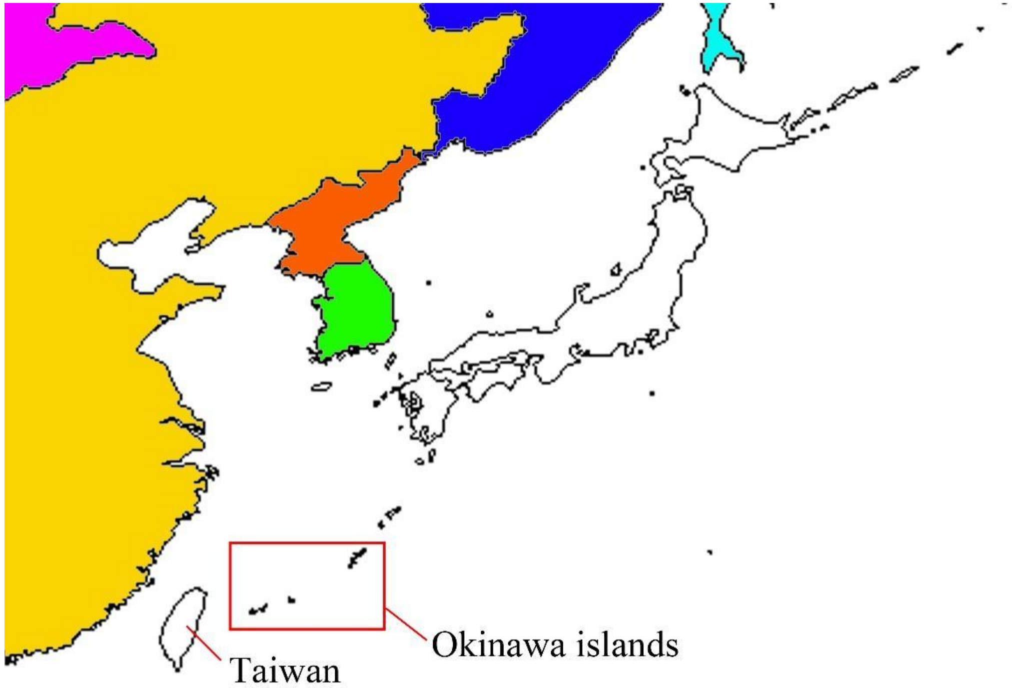

Figure S2. Map of Japan and the location of Okinawa Prefecture.

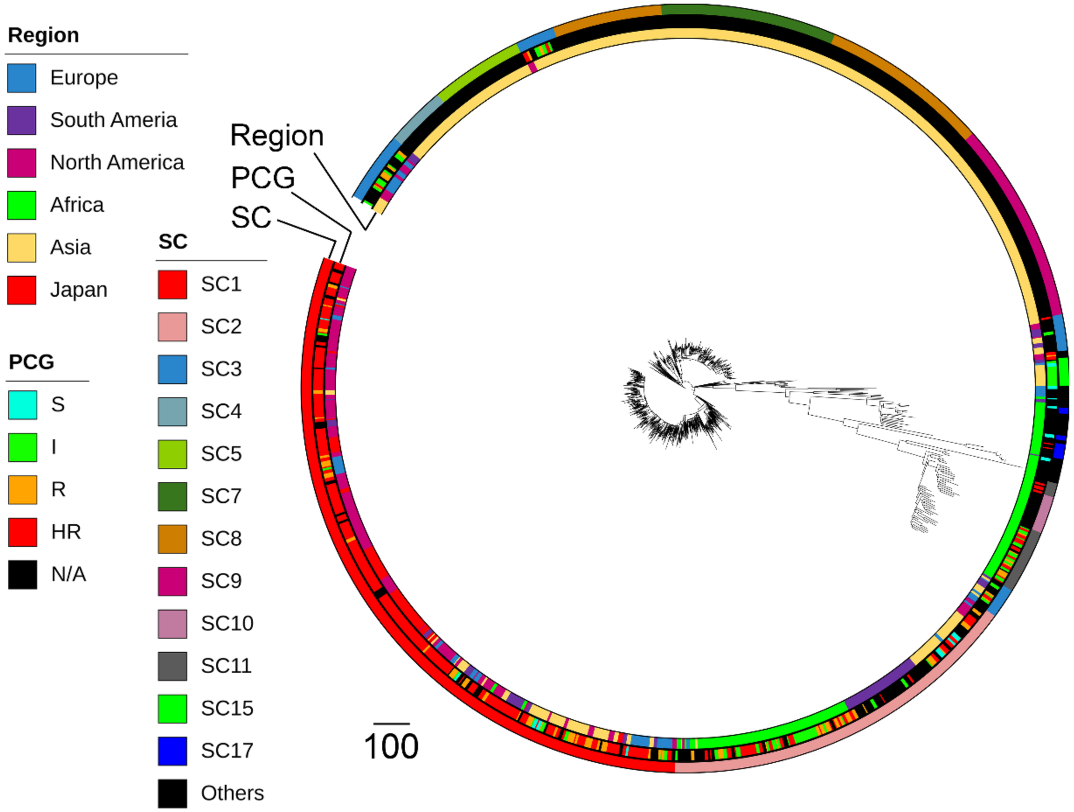

Figure S3. Phylogenetic tree of global disseminated serotype 19A-ST320 isolates created with Gubbins using *Streptococcus pneumoniae* Taiwan19F-14 as an outgroup isolate. Sequence clusters (SCs) were determined by rhierBAPS. A Middle band indicates susceptibilities of penicillin to the isolates; S: ≤0.06, I: >0.06 and ≤1, R: >1 and ≤1, HR: >2.

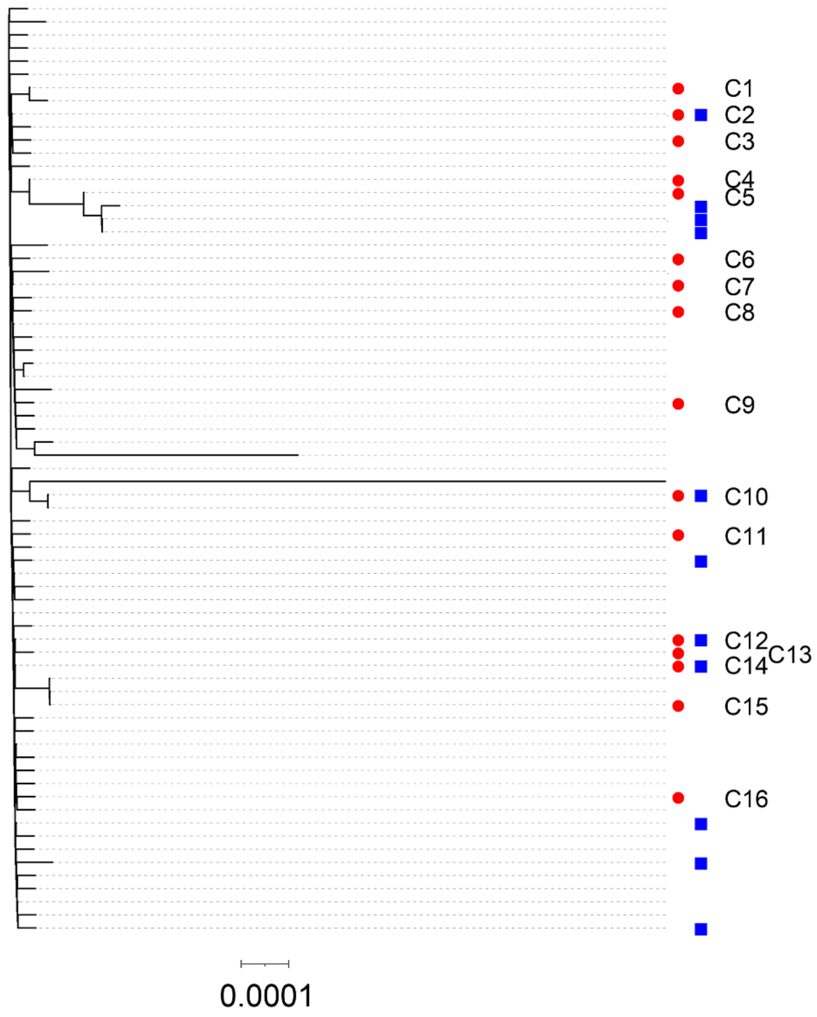

Figure S4. A phylogenetically midpoint-rooted tree reconstructed by maximum likelihood for the Tn*2010* region in SC1. Red circles and blue squares, respectively, indicate leaves that include more than one isolate and isolates from Japan.

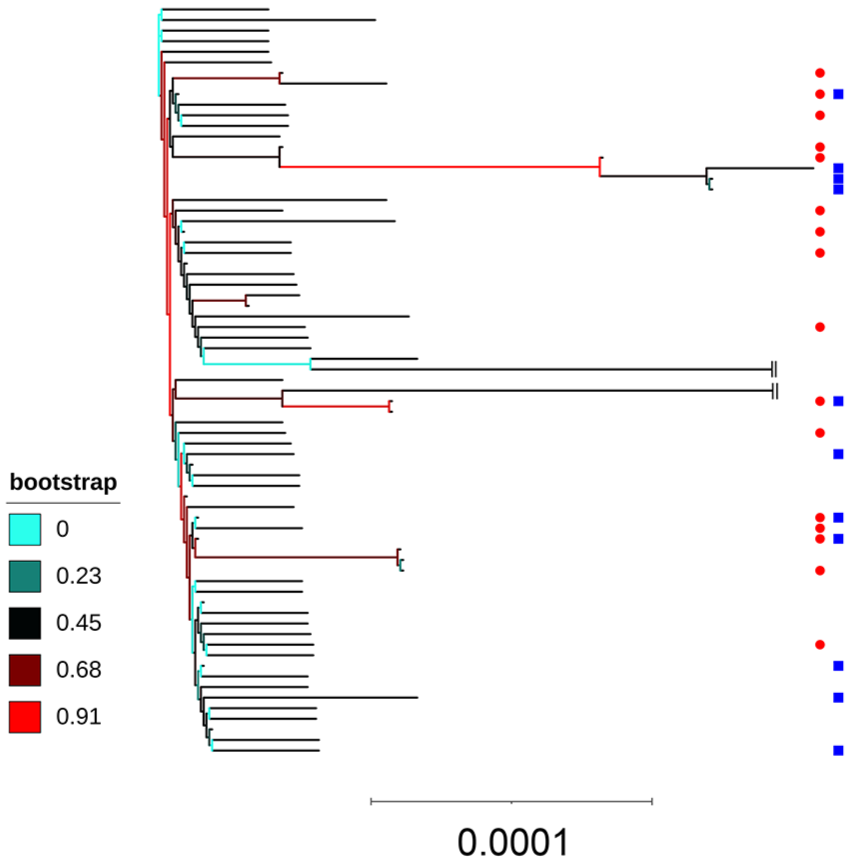

Figure S5. Magnified portion of the phylogenetically midpoint-rooted tree reconstructed by maximum likelihood for the Tn*2010* region in SC1 (Figure S3). Branch colors indicate bootstrap values. Red circles and blue squares, respectively, indicate leaves that include more than one isolate and isolates from Japan.

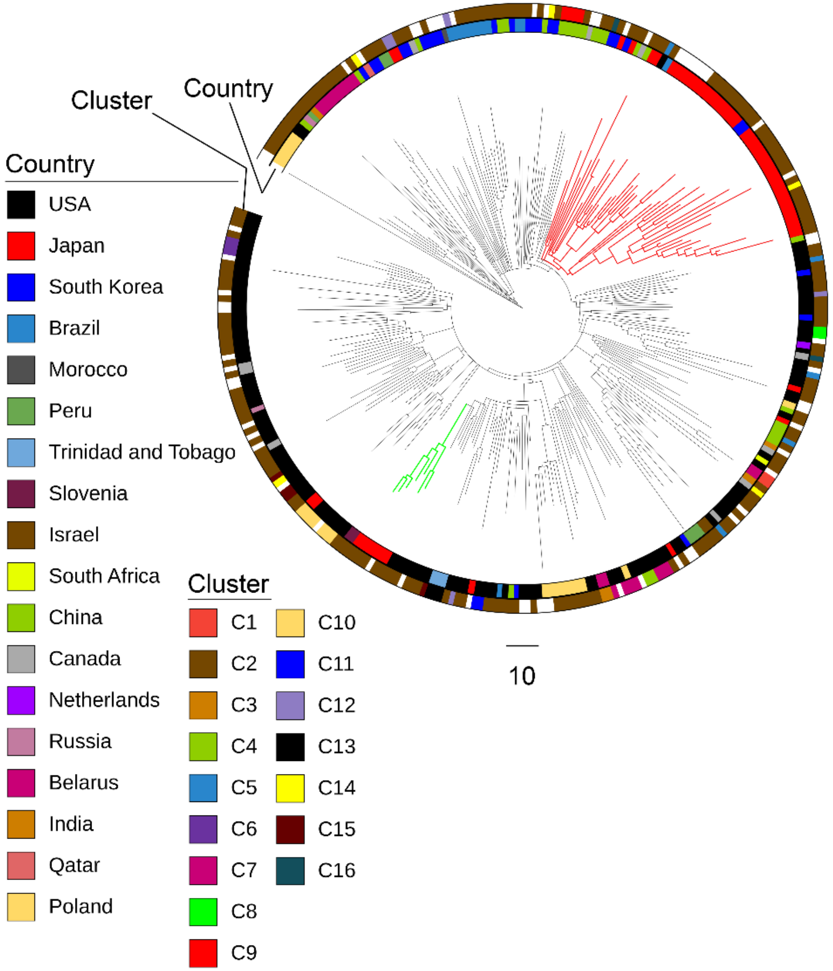

Figure S6. A phylogenetic tree of SC1 (Figure 1) with the Tn*2010* distribution. The tree was created using *Streptococcus pneumoniae* Taiwan19F-14 as an outgroup isolate. The outer circular band indicates the leaf numbers (C1-16) in Figure S4.

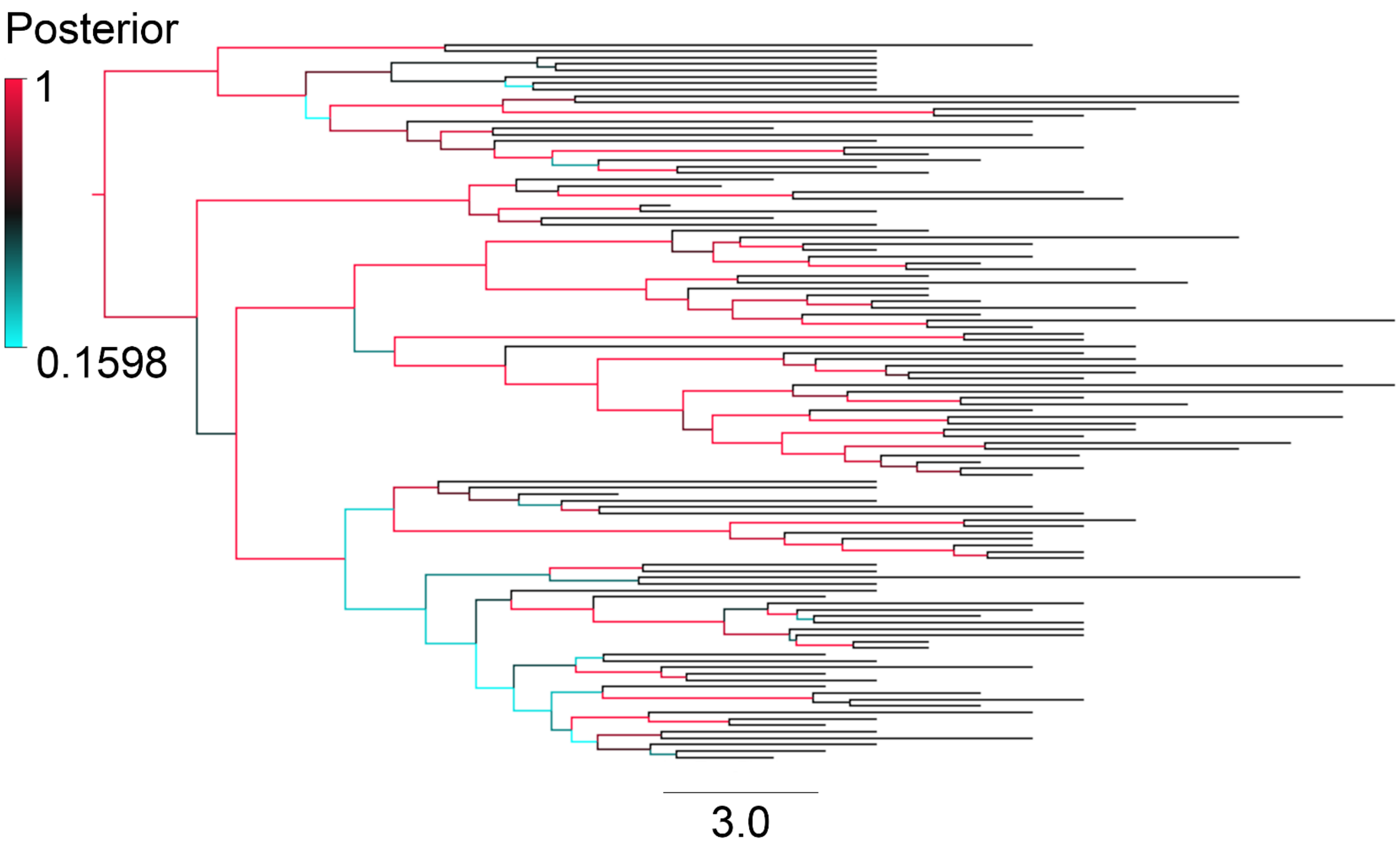

Figure S7. A maximum clade credibility tree with posteriors of each node is shown in Figure 2.

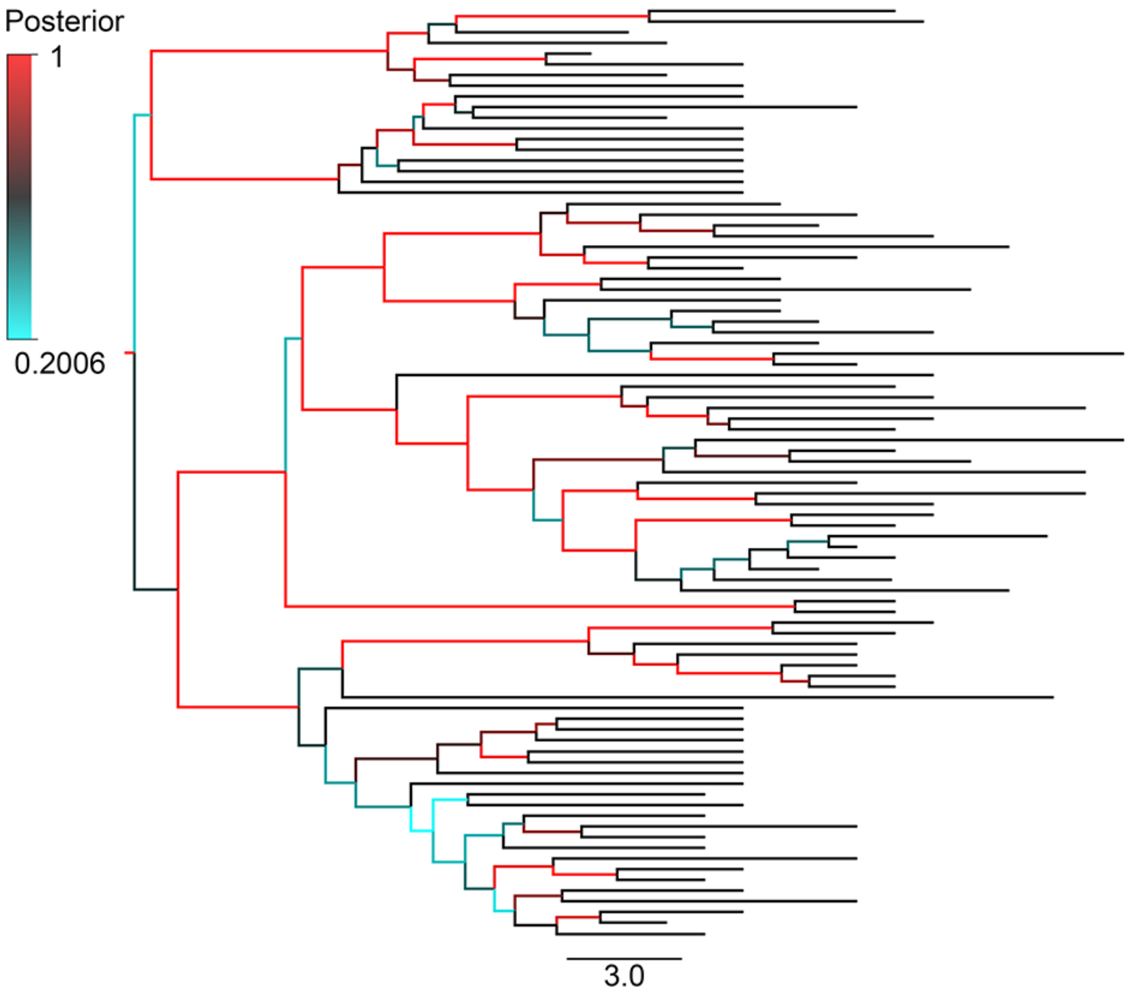

Figure S8. A maximum clade credibility tree with posteriors of each node is shown in Figure 3.

Supplementary References

1. Nakano S, Fujisawa T, Ito Y, Chang B, Suga S, Noguchi T, et al. Serotypes, antimicrobial susceptibility, and molecular epidemiology of invasive and non-invasive Streptococcus pneumoniae isolates in paediatric patients after the introduction of 13-valent conjugate vaccine in a nationwide surveillance study conducted in Japan in 2012-2014. Vaccine. 2016 Jan 02;34(1):67-76.
2. Nakano S, Fujisawa T, Ito Y, Chang B, Matsumura Y, Yamamoto M, et al. Nationwide surveillance of paediatric invasive and non-invasive pneumococcal disease in Japan after the introduction of the 13-valent conjugated vaccine, 2015-2017. Vaccine. 2019 Dec 24.
3. Suga S, Chang B, Asada K, Akeda H, Nishi J, Okada K, et al. Nationwide population-based surveillance of invasive pneumococcal disease in Japanese children: Effects of the seven-valent pneumococcal conjugate vaccine. Vaccine. 2015 Jul 31.
4. Shimbashi R, Suzuki M, Chang B, Watanabe H, Tanabe Y, Kuronuma K, et al. Effectiveness of 23-Valent Pneumococcal Polysaccharide Vaccine against Invasive Pneumococcal Disease in Adults, Japan, 2013-2017. Emerg Infect Dis. 2020 Oct;26(10):2378-86.
5. Chen S, Zhou Y, Chen Y, Gu J. fastp: an ultra-fast all-in-one FASTQ preprocessor. Bioinformatics. 2018 Sep 1;34(17):i884-i90.
6. Bankevich A, Nurk S, Antipov D, Gurevich AA, Dvorkin M, Kulikov AS, et al. SPAdes: a new genome assembly algorithm and its applications to single-cell sequencing. J Comput Biol. 2012 May;19(5):455-77.
7. Gurevich A, Saveliev V, Vyahhi N, Tesler G. QUAST: quality assessment tool for genome assemblies. Bioinformatics. 2013 Apr 15;29(8):1072-5
8. Altschul SF, Gish W, Miller W, Myers EW, Lipman DJ. Basic local alignment search tool. J Mol Biol. 1990 Oct 5;215(3):403-10.
9. Metcalf BJ, Gertz RE, Jr., Gladstone RA, Walker H, Sherwood LK, Jackson D, et al. Strain features and distributions in pneumococci from children with invasive disease before and after 13-valent conjugate vaccine implementation in the USA. Clin Microbiol Infect. 2016 Jan;22(1):60.e9-.e29.
10. Croucher NJ, Page AJ, Connor TR, Delaney AJ, Keane JA, Bentley SD, et al. Rapid phylogenetic analysis of large samples of recombinant bacterial whole genome sequences using Gubbins. Nucleic Acids Res. 2015 Feb 18;43(3):e15.
11. Li H, Durbin R. Fast and accurate short read alignment with Burrows-Wheeler transform. Bioinformatics. 2009 Jul 15;25(14):1754-60.
12. McKenna A, Hanna M, Banks E, Sivachenko A, Cibulskis K, Kernytsky A, et al. The Genome Analysis Toolkit: a MapReduce framework for analyzing next-generation DNA sequencing data. Genome Res. 2010 Sep;20(9):1297-303.
13. Walker BJ, Abeel T, Shea T, Priest M, Abouelliel A, Sakthikumar S, et al. Pilon: an integrated tool for comprehensive microbial

variant detection and genome assembly improvement. PLoS One. 2014;9(11):e112963.
14. Danecek P, Auton A, Abecasis G, Albers CA, Banks E, DePristo MA, et al. The variant call format and VCFtools. Bioinformatics. 2011 Aug 01;27(15):2156-8.
15. Tonkin-Hill G, Lees JA, Bentley SD, Frost SDW, Corander J. RhierBAPS: An R implementation of the population clustering algorithm hierBAPS. Wellcome Open Res. 2018;3:93.
16. Roberts AP, Mullany P. A modular master on the move: the Tn916 family of mobile genetic elements. Trends Microbiol. 2009 Jun;17(6):251-8.
17. Carver T, Berriman M, Tivey A, Patel C, Bohme U, Barrell BG, et al. Artemis and ACT: viewing, annotating and comparing sequences stored in a relational database. Bioinformatics. 2008 Dec 1;24(23):2672-6.
18. Darriba D, Posada D, Kozlov AM, Stamatakis A, Morel B, Flouri T. ModelTest-NG: a new and scalable tool for the selection of DNA and protein evolutionary models. Mol Biol Evol. 2019 Aug 20.
19. Kozlov AM, Darriba D, Flouri T, Morel B, Stamatakis A. RAxML-NG: a fast, scalable and user-friendly tool for maximum likelihood phylogenetic inference. Bioinformatics. 2019 Nov 1;35(21):4453-5.
20. Drummond AJ, Rambaut A. BEAST: Bayesian evolutionary analysis by sampling trees. BMC Evol Biol. 2007 Nov 8;7:214.
21. Page AJ, Taylor B, Delaney AJ, Soares J, Seemann T, Keane JA, et al. SNP-sites: rapid efficient extraction of SNPs from multi-FASTA alignments. 2016 2016/04/29.
22. Rambaut A, Lam TT, Max Carvalho L, Pybus OG. Exploring the temporal structure of heterochronous sequences using TempEst (formerly Path-O-Gen). Virus Evol. 2016 Jan;2(1):vew007.
23. Lemey P, Rambaut A, Drummond AJ, Suchard MA. Bayesian phylogeography finds its roots. PLoS Comput Biol. 2009 Sep;5(9):e1000520.
